# Cloning and Characterization of Maize miRNAs Involved in Responses to Nitrogen Deficiency

**DOI:** 10.1371/journal.pone.0029669

**Published:** 2012-01-03

**Authors:** Meng Zhao, Huanhuan Tai, Suzhen Sun, Fusuo Zhang, Yunbi Xu, Wen-Xue Li

**Affiliations:** 1 Key Laboratory of Plant and Soil Interactions, Ministry of Education, China, and College of Resources and Environmental Sciences, China Agricultural University, Beijing, China; 2 Institute of Crop Science, National Key Facilities for Crop Genetic Resources and Improvement, Chinese Academy of Agricultural Sciences, Beijing, China; 3 International Maize and Wheat Improvement Center (CIMMYT), Carretera, Mexico-Veracruz, EI Batan, Texcoco, Mexico; French National Center for Scientific Research - Institut de biologie moléculaire et cellulaire, France

## Abstract

Although recent studies indicated that miRNAs regulate plant adaptive responses to nutrient deprivation, the functional significance of miRNAs in adaptive responses to nitrogen (N) limitation remains to be explored. To elucidate the molecular biology underlying N sensing/signaling in maize, we constructed four small RNA libraries and one degradome from maize seedlings exposed to N deficiency. We discovered a total of 99 absolutely new loci belonging to 47 miRNA families by small RNA deep sequencing and degradome sequencing, as well as 9 new loci were the paralogs of previously reported miR169, miR171, and miR398, significantly expanding the reported 150 high confidence genes within 26 miRNA families in maize. Bioinformatic and subsequent small RNA northern blot analysis identified eight miRNA families (five conserved and three newly identified) differentially expressed under the N-deficient condition. Predicted and degradome-validated targets of the newly identified miRNAs suggest their involvement in a broad range of cellular responses and metabolic processes. Because maize is not only an important crop but is also a genetic model for basic biological research, our research contributes to the understanding of the regulatory roles of miRNAs in plant adaption to N-deficiency stress.

## Introduction

As the key component of many macromolecules, including proteins and nucleic acids, nitrogen (N) is essential for the normal growth and development of plants. Because an insufficient N supply in soil can limit crop productivity, substantial research has been directed at improving the N-use efficiency by plants. The morphological, physiological, and biochemical adaptations to the variations of N availability in soils are at least partially dependent on changes in gene expression [1]. Several global expression profiling analyses have revealed that N-responsive genes are involved in a wide range of processes, such as primary and secondary metabolism, protein synthesis, cytokinin translocation and signaling, auxin transport and signaling, and transcription regulation [2], [3]. *AtANR1* was the first transcription factor identified that controlled nutrient-induced changes in root architecture [4]. Other transcription factors involved in an N-stress responsive pathway contained *GNC* (GATA, nitrate-inducible, carbon metabolism-involved), *DOF1* (DNA binding with one finger), and *CCA1* (Circadian Clock Associated 1) [5]–[7]. Despite this substantial progress, the molecular basis underlying N sensing/signaling remains incompletely understood.

In recent years, small regulatory RNAs have attracted attention because of their important roles in posttranscriptional or translational gene regulation [8]–[12]. MicroRNAs (miRNAs) and small interfering RNAs (siRNAs) are the two major groups of small RNAs in plants that serve as negative regulators of gene expression. miRNAs are processed from hairpin precursors by the ribonuclease III-like enzyme Dicer Dicer-like1 (DCL1) or DCL4. Unlike miRNAs, siRNAs are generated from perfectly complementary, long, double-stranded RNAs by RNA -dependent RNA polymerase 6 (RDR6)/RDR2. Plant miRNAs regulate a broad range of physiological processes, such as flowering time [13], leaf morphogenesis [14], nodule development [15], and adaption to abiotic stresses [16], [17].

Recent findings have also revealed the important roles of plant miRNAs in nutrient deficiency. For example, miR395 regulates sulfur assimilation and translocation by adjusting the mRNA levels of ATP sulfurylase and a low affinity sulfur transporter [18], [19]. miR399 regulates phosphate homeostasis in *Arabidopsis* through the suppression of a ubiquitin-conjugating E2 enzyme, PHO2 [20], [21]. miRNAs might also play a role in plant responses to N stresses. Gifford et al. reported that miR167 in pericycle cells mediated lateral root initiation and growth in response to nitrate [22]. By analysis of primary transcripts of known miRNAs, several N-responsive miRNAs in *Arabidopsis* were found and their abundances were strongly dependent on P or N status in the phloem sap of rapeseed (*Brassica napus*), suggesting that these miRNAs might act as systemic signals [23]. Our results also showed that miR169 was strongly down-regulated by N starvation, and transgenic *Arabidopsis* plants overexpressing *MIR169a* accumulated less N and were more sensitive to N stress than the wild type [24].

As an important model system for basic biological research, maize has contributed significantly to our knowledge of plant development and evolution, and this understanding has been used to elucidate the regulatory functions of miRNAs [25]. The total number of miRNAs in an individual organism was estimated to represent ∼1% of the total number of coding genes [16], [26]. Gene content in the maize B73 whole genome assembly was estimated at between ∼37,000 and ∼39,000. So far, only 150 high-confidence genes within 26 miRNA families have been reported in maize [25], and the number of miRNAs identified in maize was far from saturation. The two main approaches to identify new miRNAs have been advanced high-throughput sequencing technologies and bioinformatic analysis. Bioinformatic analysis based on sequence similarity effectively predicted conserved miRNAs among different species independent of their abundance or spatial/temporal expression patterns [18]. However, many miRNAs are species-specific and have evolved only recently. Advanced high-throughput sequencing technologies has revealed non-conserved miRNAs in various plant species [16], [27]–[31]. Because the methods are reproducible, advanced high-throughput sequencing technologies were also used to analyze small RNA profiles, in which the normalized small RNA reads from different tissues or developmental stages were compared [32], [33].

In the present research, we aimed to identify new miRNAs and discover N-deficiency regulated miRNAs in maize by using a deep sequencing approach. We constructed not only four small RNA libraries from N-deficient and control maize seedlings, but also a degradome from N-deficient roots. Through library sequencing and analysis, we discovered a total of 99 absolutely new loci belonging to 47 miRNA families by small RNA deep sequencing and degradome sequencing, as well as 9 new loci were the paralogs of previously reported miR169, miR171, and miR398. The results of small RNA northern blot analyses showed that the expressions of miR169s, miR395s, miR528s, miR827s, miRC1, miR169w, miR171p/q, miRC19 and miRC37 were downregulated or upregulated by N limitation. Our results contribute to the understanding of the regulatory roles of miRNAs in plant adaption to N- deficiency.

## Results

### Analysis of small RNA library data sets in response to N deficiency

For identification of miRNAs and other endogenous small RNAs in response to N deficiency, four small RNA libraries from N-sufficient roots (R+N), N-deficient roots (R−N), N-sufficient shoots (S+N), and N-deficient shoots (S−N) were generated and sequenced by Solexa high-throughput sequencing technology. These four small RNA libraries yielded a total of more than 35 million raw reads, and approximately 90% of the raw reads remained after 3′ adaptor trimming (Table S1). Of these, more than 24 million reads could be perfectly mapped to maize B73 RefGen_V2 (http://archive.maizesequence.org/index.html). Sequences that could not be mapped to the maize genome were discarded, and only those perfectly mapped were analyzed further. The genome-matched sequences were classed into several RNA groups, including known miRNA, rRNA, tRNA, snRNA, snoRNA and others (Table S2). The known miRNAs accounted for 27.9, 46.4, 28.7, and 33.9% of the small RNA libraries for S+N, S−N, R+N, and R−N, respectively. However, the known miRNAs represented only ∼0.1% of the total number of unique sequences in the shoot small RNA libraries, and even a lower percentage of the unique sequences in the root small RNA libraries (0.08% for R+N small RNA library, 0.05% for R−N small RNA library). The highest proportion of unique sequences was unclassified small RNA sequences, which might include novel miRNA candidates.

**Figure 1:**
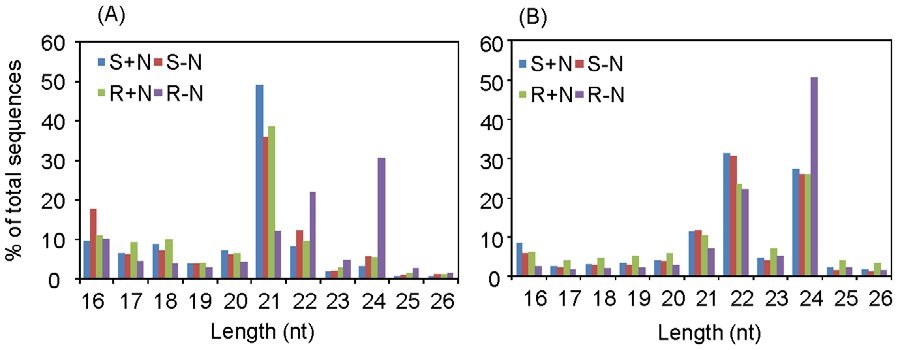
Size distribution of redundant (A) and unique (B) small RNA sequences. nt, nucleotides.

The cloning frequency of different sized small RNAs (16–26 nt) was similar between S+N and S–N libraries. Consistent with the previous report by Pantaleo et al. [34], 21-nt small RNAs in total sequence reads in S+N, S–N, and R+N libraries were dominant (Figure 1A). In contrast, two major peaks at 24 nt and 22 nt were observed in the R−N library (Figure 1A). When the unique sequence signatures were examined, the 24- and 22- nt class of small RNAs were most abundant, and most of them appeared only once in corresponding small RNA library data sets (Figure 1B), suggesting that they are diverse in maize.

### Identification of novel miRNAs from N-deficient maize by small RNA library sequencing

Confident annotation of novel species-specific miRNAs requires *dcl1* or *dcl4* knockout mutants [35], [36]. In the absence of these mutants, one of the most important criteria is the sequencing of miRNA* [37]. Although 953 novel loci with miRNA precursor-like stem-loop structures were found in our small RNA library data sets, only 102 loci belonging to 46 miRNA families were annotated as completely new miRNAs based on the criteria (Table S3). Many of newly identified miRNAs contained 21–22 nt and 17 of the 46 novel miRNA families began with a 5′ uridine. Twenty of these candidate miRNAs had different independent genomic locations (e.g. miRC34 had 13 different genomic loci), and others had a unique genomic location. The folding free energies ranged from −21.6 to −128.54 kcal mol^−1^ with an average of −64.10 kcalmol^−1^; this average was similar to the −72.4 kcal mol^−1^ in wheat and −71.0 kcal mol^−1^ in rice, and was much lower than that of tRNA and rRNA [38]. In maize, a subset of miRNAs was organized as compact clusters, with fewer than 2000 nt separating adjacent genes [25]. In the present research, all the novel MIRs we have identified existed as tandem paralog clusters (Table S3).

**Figure 2:**
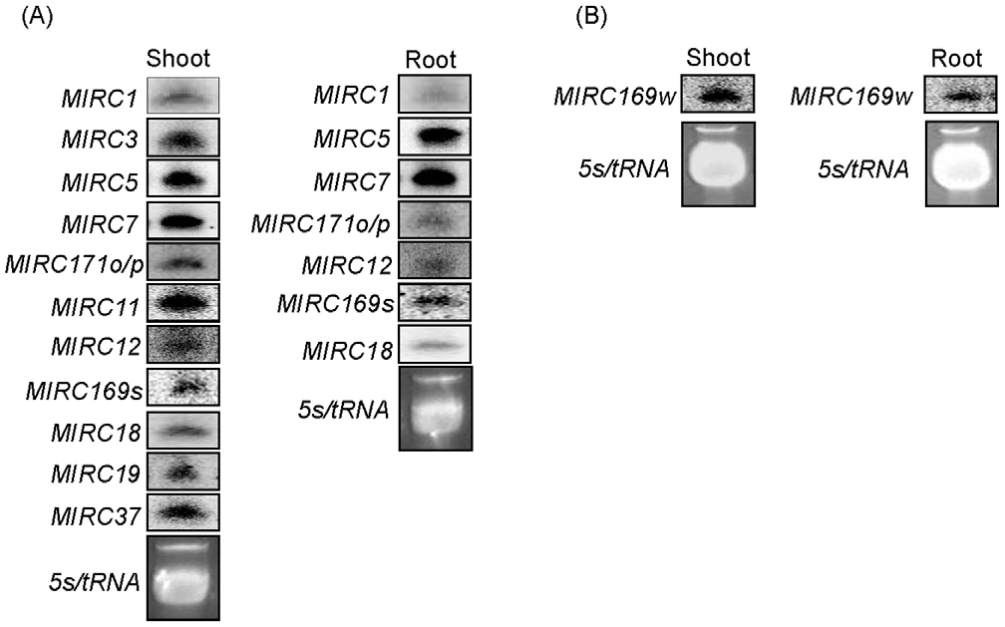
Detection of novel miRNA candidate identified by small RNA library (A) or degradome (B) sequencing. Plants were grown hydroponically with full-strength Hoagland's nutrient solution for 2 weeks and then transferred to the N-free or N-replete medium for 2 days before small RNA was isolated from leaves and roots. Five micrograms of small RNA from each sample was loaded and hybridized with corresponding ^32^P-labeled probes. 5s/tRNA is shown as a loading control.

Among the novel miRNAs, four families belonged to known miRNAs based on their homology with known miRNAs or miRNA precursors reported in maize or other plant species. miRC8, which had two independent genomic loci in maize, and miRC31 were the homologues of zma-miR171. miRC16, miRC27 (three different genomic loci), and miRC24 were the orthologs of zma-miR169 and zma-miR398, respectively. Thus, we re-designated them as zma-miR171o, zma-miR171p, zma-miR171q, zma-miR169s, zma-miR169t, zma-miR169u, zma-miR169v, and zma-miR398c. The other novel miRNAs were not found in other plant species, indicating that they are maize-specific. The non-conserved miRNAs were usually expressed at a low level. Although miRC37 was abundant in the S−N library, the sequence reads of most of the novel miRNAs in the four libraries were under 100 TPQ (Table S4). miRC8 was abundantly expressed in maize shoots, while miRC4 showed elevated expression in roots. miRC34, miRC35, miRC36, miRC37, and miRC38 were only observed in the S−N library, whereas miRC42, miRC43, miRC44, miRC45, and miRC46 were distinctly abundant in N-limited root (Table S4), suggesting that the expression patterns of novel miRNAs were tissue-specific or stress-regulated. To further demonstrate that the novel miRNAs were authentic, we chose candidate miRNA sequences with at least five reads for small RNA northern blot analysis and a total of 15 probes (six from shoots, one from roots, and eight from both shoots and roots) were selected. Except miRC4, miRC23, and miRC26 from roots, miRNAs with around 21 nt could be clearly detected using small RNA blot analysis (Figure 2A), further demonstrating the authenticity of the novel miRNAs that we identified in our four libraries.

### Identification of novel miRNAs from N-deficient maize roots by degradome sequencing

The abundance of miRNA* is usually low. Even for conserved miRNAs, the miRNA* could not be detected by small RNA deep sequencing sometimes; this was the case with miR394 in the current research (data not shown). To continue with the identification of the potentially novel miRNAs, we constructed a degradome using total RNA from R−N for parallel analysis of RNA ends (PARE), which can be used to predict novel miRNAs that mediate mRNA cleavage [39]. We obtained a total of 15,328,555 reads, corresponding to 30,638 unique sequences perfectly matching the maize genome. Based primarily on the secondary structures of the miRNA candidates and their abundances in the R−N library, we identified six other novel loci belonging to five families (Table 1). Four of the five novel miRNA families began with a 5′ uridine. The folding free energies ranged from −41.2 to −96.0 kcal mol^−1^ with an average of −65.49 kcal mol^−1^. Among the novel miRNAs, miRC47 was the homologue of zma-miR169 and re-designated as zma-miR169w. The newly identified zma-miR169w was much more abundant than the conserved zma-miR169, whereas the abundances of miRC48–51 were relative low (Table S4). Thus, we selected zma-miR169w and miRC50, whose abundances were relatively high, for small RNA northern blot analysis; and we clearly detected the expression of zma-miR169w, but not of miRC50 (Figure 2B).

**Table 1:** Sequences of novel miRNAs identified by the degradome sequencing in maize.

miRNA	Sequence	Lengthnt	Chr	Start	End
**miRC47** **miR169w**	UAGCCAAGGAUGAGCUGCCUG	21	2	189774689	189774920
miRC48	UUCUUAGGAAAAGAGGUCGGC	21	8	15615633	15615772
miRC49	AAUCCGUGAUGCAAGACAAUU	21	3	122633333	122633582
miRC50	UGCUGUGAUGAAGAUGCUCA	20	9	143934647	143934803
miRC51	UCCAAGGAAGACACUCGGCAAA	22	3	117417189	117417259
		22	7	72813292	72813363

New paralogs of pre-existing miRNA families were cross-referenced and highlighted.

### Expression profiling of conserved miRNAs in response to N limitation

**Figure 3:**
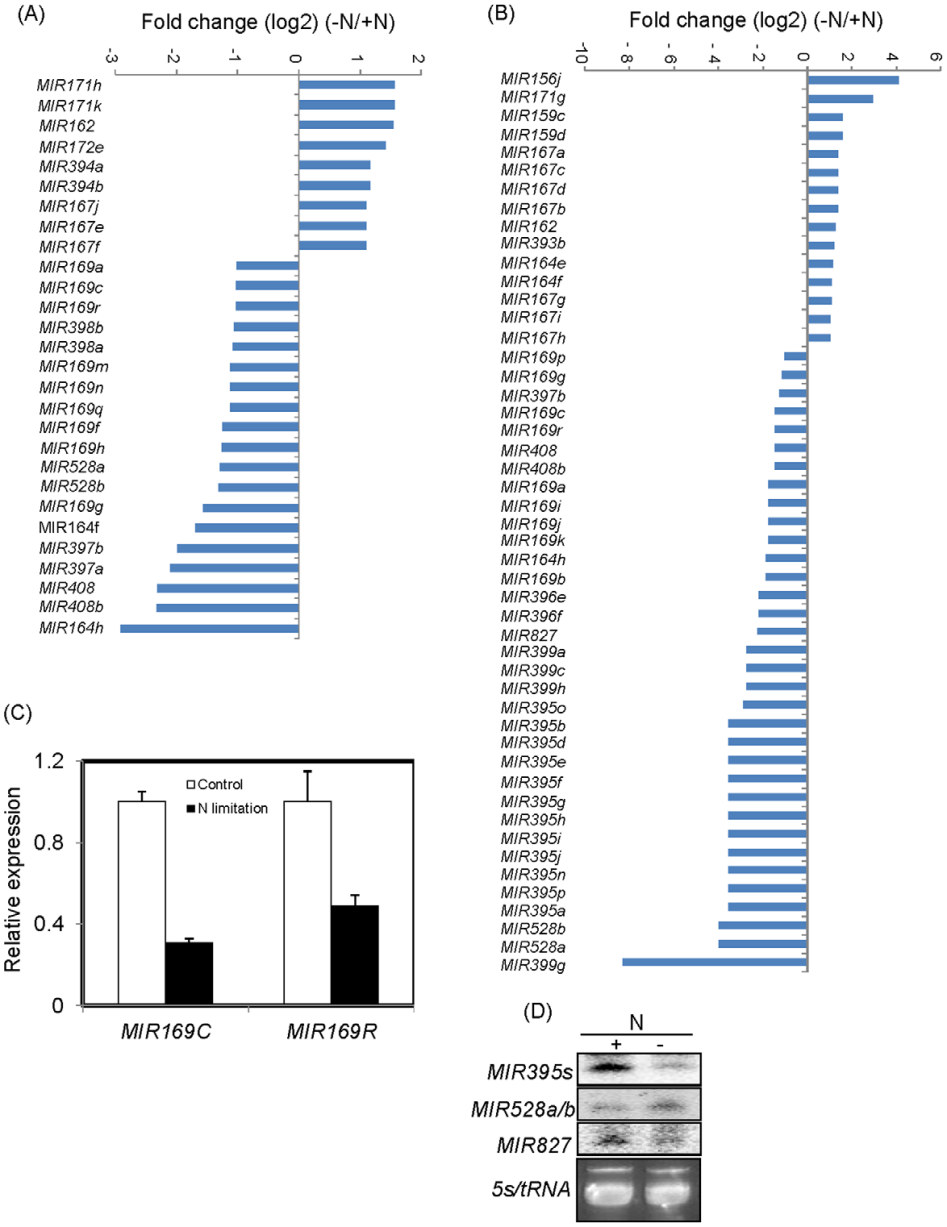
Differential expression of conserved miRNAs in response to N deficiency in shoots (A) and roots (B). Only miRNA genes with > 2-fold relative change are shown. Selected miRNAs from roots were validated by Real time RT-PCR (C) or small RNA northern blot (D). Maize seedlings were grown hydroponically with full-strength Hoagland's nutrient solution for 2 weeks and then transferred to the N-free or N-sufficient medium for 2 d before total/small RNA was isolated. Real-time RT-PCR quantifications were normalized to the expression of *ZmGAPC2*. The results represent SD of three replicates. A 40 µg small RNA was loaded per lane and hybridized with corresponding ^32^P-labeled probes. 5s/tRNA is shown as a loading control.

Computational approaches based on homology and secondary structure have identified 150 genes within 26 miRNA families in maize [25]. Except for zamiR482, all of the other 25 annotated miRNA families were detected in our small RNA library data sets (Table S5). Twenty-eight and 49 conserved miRNAs with > 2-fold relative change were identified in shoots and roots, respectively, based on the difference in numbers of normalized sequence reads between N deficient and N sufficient tissues (Figure 3A and B). The downregulation of miR169 caused by N deficiency in both shoots and roots agreed with our previous reports on *Arabidopsis* (Figure 3A and B), indicating the reliability of our sequencing data. In shoots, the expression of *MIR162*, *MIR167s*, and *MIR394s* was upregulated by N deficiency, whereas the expression of *MIR169s*, *MIR397s*, *MIR398s*, *MIR408s*, and *MIR528s* was significantly downregulated by N deficiency (Figure 3A). *MIR162* and *MIR167s* were also upregulated by N deficiency in roots, and the expressions of *MIR169s*, *MIR397s*, *MIR408s*, *MIR528s*, *MIR395s*, and *MIR827* were clearly decreased in N -deficient roots (Figure 3B).

Because of the importance of roots in the sensing and uptake of nutrients, we selected *MIR169c/r*, *MIR395a/b/d/e/f/g/h/i/j/n/p*, *MIR528a/b*, and *MIR827* for further validation by Real-time RT PCR or small RNA northern analysis (Figure 3C and D). The expression of *MIR169a/b*, *MIR395a/b/d/e/f/g/h/i/j/n/p*, and *MIR827* was substantially suppressed by N deficiency, which was consistent with the sequencing data. The only exception was *MIR528a/b*, whose expression was upregulated by N deficiency in the northern blot analysis but downregulated in the sequencing data.

### Discovery of novel N-responsive miRNAs

**Figure 4:**
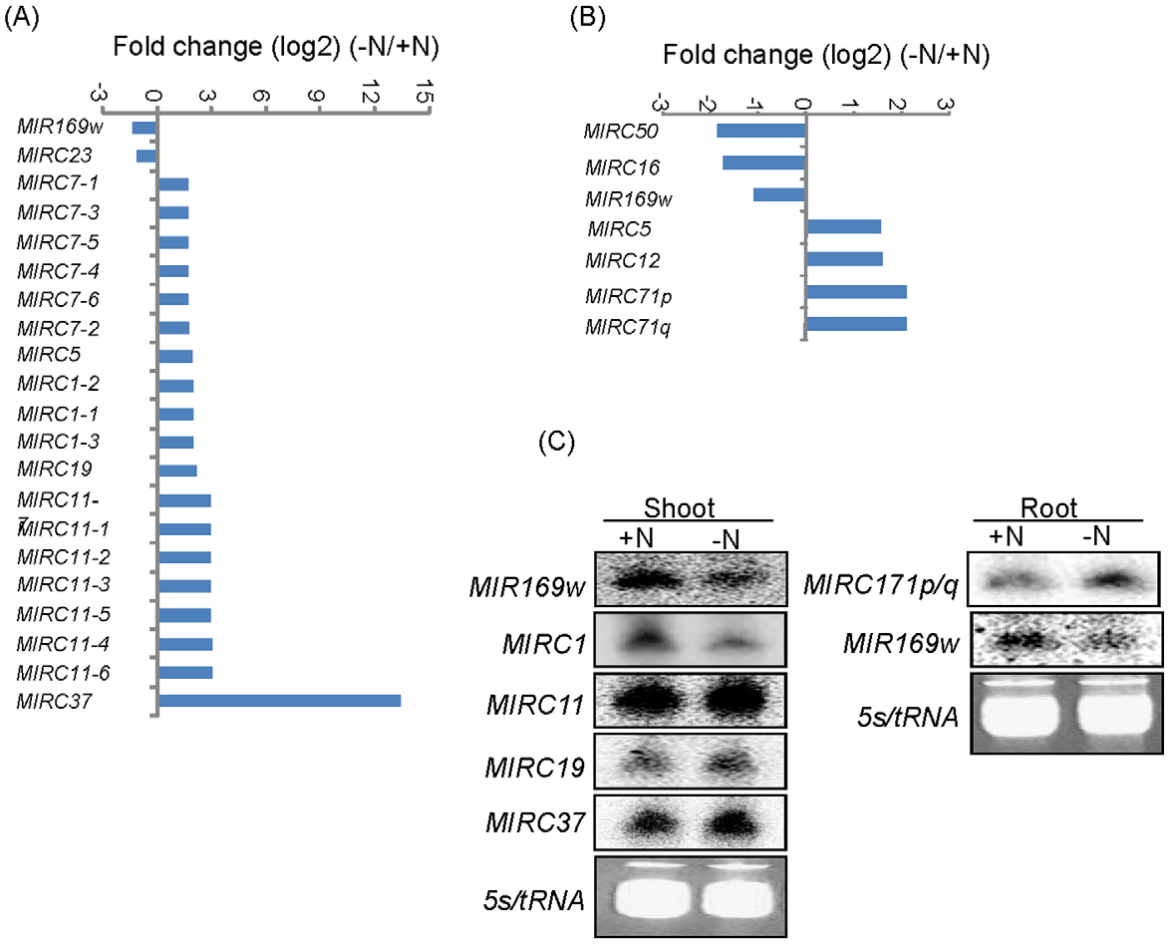
Differential expression of newly identified miRNAs in response to N deficiency in shoots (A) and roots (B). Only miRNA genes with > 2-fold relative change were shown. Selected miRNAs from shoots and roots were validated by small RNA northern blot (C). A 40 µg small RNA was loaded per lane and hybridized with corresponding ^32^P-labeled probes. 5s/tRNA is shown as a loading control.

The newly identified zma-miR169s, zma-miR169t, zma-miR169u, and zma-miR169v (identified by small RNA deep sequencing) and zma-miR169w (identified by degradome) were also downregulated in both shoots and roots by N deficiency (Table S4), which was consistent with the conserved zma-miR169, suggesting again the reliability of the sequencing data. To improve accuracy, we selected only the newly identified miRNAs with sequence reads over five TPQ in at least one library for further analysis. Twenty-one miRNAs in eight families in shoots and seven miRNAs in six families in roots with > 2-fold relative change were identified (Figure 4A and B). The expression of the novel miRNAs with greater than 4-fold relative change and miR169w was validated by small RNA northern blots. Seven families of 11 novel N-responsive miRNAs, including miRC1, miRC11, miR171p/q, miRC16, miRC19, miRC37 and miR169w, were selected (Figure 4C). miR171p/q and miR169w exhibited an expression pattern perfectly matching the sequence frequency in the libraries. miRC19 and miRC37 were slightly upregulated, and miRC1 was significantly downregulated by N deficiency. The discrepancy between northern blot analysis and sequencing data might be due to the insufficient sequence reads of the newly identified miRNAs, which has been also reported by Li et al. [40], or normalization in this case could not be simply done by adjusting reads to TPQ.

### Prediction and validation of targets of newly identified miRNAs

To predict the potential targets of the novel identified miRNAs, we searched the Filtered Gene Set transcripts (Release 4a.53) of the B73 maize genome sequenced for maize mRNAs with sequence complementarity to the miRNAs according to the procedure and criteria described by Allen et al. [41] and Schwab et al. [42]; we also searched the results of the degradome sequencing for targets. For the potential targets of novel miRNAs identified by small RNA deep sequencing, the mispair score and minimum free energy (MFE) ratio limits to ≤4 and ≥0.70 were applied. Our analysis predicted 80 unique potential genes targeted by 31 miRNA sequences belonging to 20 families (Table S6). We also detected 28 unique targets for novel miRNAs identified by the degradome (Table 2). That the others lacked predicted targets might be due to the recent development/appearance of these miRNAs or to unannotated mRNAs in the maize genome.

**Table 2:** Predicted targets of novel miRNAs identified by the degradome sequencing in maize.

miRNA	Predicted targets	Putative function of targets
miR169w	GRMZM2G000686_T01; GRMZM2G000686_T02GRMZM2G000686_T03; GRMZM2G000686_T04GRMZM2G000686_T05; GRMZM2G000686_T06GRMZM2G000686_T07; GRMZM2G000686_T08 GRMZM2G000686_T09; GRMZM2G000686_T10GRMZM2G165488_T01; GRMZM2G165488_T02GRMZM2G165488_T03; GRMZM2G165488_T04GRMZM2G091964_T01; GRMZM2G091964_T02GRMZM2G091964_T03	Nuclear transcription factor Y subunit
miRC48	GRMZM2G134797_T01; GRMZM2G134797_T02	ATP binding/nucleoside diphosphate kinase
miRC49	GRMZM2G006953_T02; GRMZM2G006953_T03	Nuclear transport factor, putative
miRC50	GRMZM2G121820_T01; GRMZM2G121820_T02	Brassionsterioid Insensitive 1-associated receptor kinase 1 precursor, putative
miRC51	GRMZM2G054300_T01; GRMZM2G054300_T02GRMZM2G054300_T03; GRMZM2G054300_T04GRMZM2G054187_T01	L-ascorbate peroxidase;

In agreement with previous reports for *Arabidopsis* [16], [43], our predicted target sites fell in the open reading frames (ORFs). Interestingly, three miRNAs (miRC11, miRC37, and miRC42) had been predicted to target the ORFs of GRMZM2G030823_T01, GRMZM2G030823_T02 and GRMZM2G030823_T03. Unlike the conserved miRNAs whose targets showed a strong preference for transcription factor activity or transcription regulatory activity [25], most of the newly identified miRNAs were predicted to target mRNAs encoding important enzymes including protein kinases, zinc finger family protein, and oxidoreductase (Table 2 and Table S6). To further understand the function of the predicted novel miRNA target genes, we investigated target enrichment in Gene Ontology [44]. The newly identified targets were enriched in binding activity, signal transducer activity, transmembrane transporter activity, and kinase activity (data not shown), indicating that these novel miRNAs might play an important role in metabolic processes and signaling transduction pathways.

**Figure 5:**
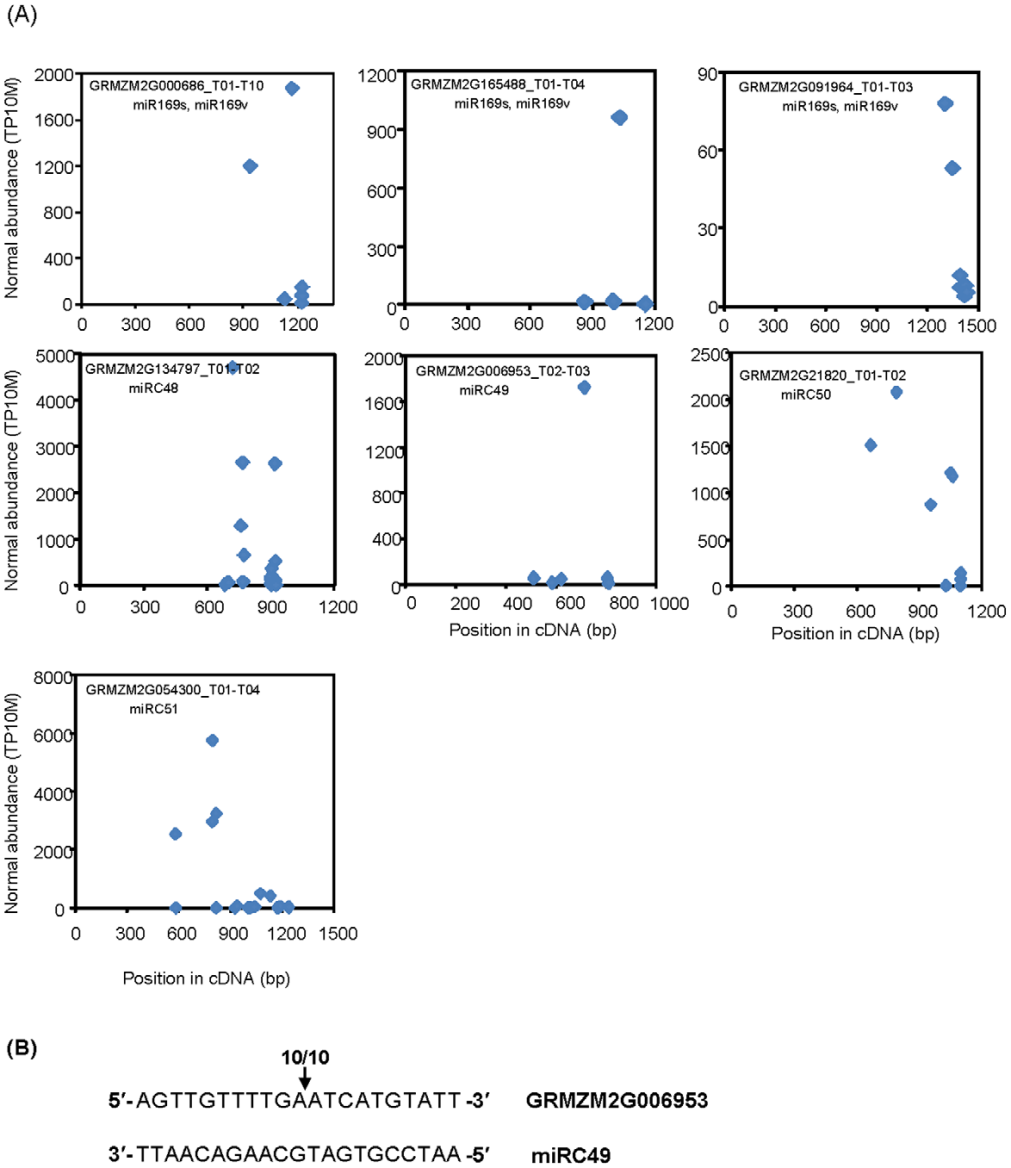
Target plots of validated miRNA targets. A degradome cDNA library was constructed from N-deficient roots and subjected to Illumina sequencing. The abundance of each signature was plotted as a function of its position in the transcript (A). The cleavage sites were further confirmed for two selected targets by 5′ RACE (B). The number above the vertical arrows indicate the data from 5′ RACE confirmation.

To verify targets of the miRNAs that were newly identified by small RNA sequencing, we used the high-throughput degradome sequencing approach and the total RNA from N-deficient root. For conserved miRNAs, we identified a total of 72 target mRNAs that had at least one degradome tag matching the corresponding cleavage sits, for *MIR166a/b/c/d/g/h/I*, *MIR169s*, *MIR172b/c/d*, *MIR395s*, *MIR408*, and *MIR408b* (data not shown). We also detected the potential targets of miR164f*, miR167g*/h*/i*, miR169r*, miR168a*/b* and miR827* by the degradome (Table 3), suggesting that miRNA* also mediated mRNA cleavage. For the novel miRNAs (*MIR169w*, *MIRC48*, *MIRC49*, *MIRC50*, and *MIRC51*) identified by the degradome, we plotted the abundance of each signature as a function of its position in the transcript targets as described by German et al. [39]. Only the two targets of zma-miR169s were detected (Figure 5A). The low identification frequency might be due to the low abundance of these miRNA and/or the different spatial distribution (miRC27–28 were only detected in the N-sufficient condition; miRC34–38 and miR39–41 were found only in the S−N library and the R+N library, respectively).

**Table 3:** miRNA* mediated mRNA cleavage detected by degradome sequencing in maize.

miRNA*	Predicted targets	Putative function of targets
miR164f*	GRMZM2G308687_T01(982)	Glutathione transferase
miR167g*	GRMZM2G032315_T01(609); GRMZM2G032315_T03(615)	60S acidic ribosomal protein P1
miR167h*/I*	GRMZM2G113414_T01(382); GRMZM2G113414_T02(515)	Protein translation factor SUI1
miR168a*	GRMZM2G369839_T01(482)	Putative uncharacterized protein
miR168b*	GRMZM2G369839_T01(482)	Putative uncharacterized protein
miR169r*	GRMZM5G818977_T03(903);GRMZM5G818977_T04(908)	Putative uncharacterized protein
miR827*	GRMZM2G164325_T01(977);GRMZM2G164325_T02(1345)	Putative uncharacterized protein

The gene GRMZM2G006953, encoding a putative nuclear transport factor 2, was predicted to be a target of miRC49. To further confirm the targets solely proven by their target plots, the cleavage of the mRNA of GRMZM2G006953 was confirmed by 5′ RACE using RNA from N-deficient roots (Figure 5B). The cleavage of GRMZM2G053779, a target of miR408 (conserved miRNA) was also experimentally validated by 5′ RACE (data not shown). The mapped cleavage sites of both conserved and novel mirNAs were in agree with those identified by degradome, indicating the reliability of degradome sequencing data.

## Discussion

To obtain high crop yields, farmers have greatly increased the application of N fertilizer, which has resulted in the severe environmental degradation [45]. Recently, substantial research has been directed at improving the N-use efficiency of plants, and our previous results clearly demonstrated that miR169 was involved in N-deficiency responses in *Arabidopsis* [24]. This motivated us to identify N-responsive miRNAs. Pant et al. detected some N status-responsive pri-miR species by a quantitative real-time polymerase chain reaction platform [23]; however, the data came from dicotyledon, *Arabidopsis*, and this technology could not find non-conserved miRNAs. The present study focused on maize, a valuable graminaceous monocot that provides feed, fodder, and biofuel. By deep sequencing four small RNA libraries and one degradome from maize seedlings exposed to N deficiency, we were able to add 47 new miRNA families to the previously published 26 families in maize. In addition, we identified three, five, and one new member for the families of miR171, miR169, and miR398, respectively (Table 1 and Table S3).

The detection of miRNA* has been considered one of the most important criteria for miRNA identification. In the miRNA/miRNA* duplex, the mature miRNA is selected to load into AGO protein and miRNA* is degraded. Thus, the abundance of miRNA* is rather low. In our deep sequencing data, several miRNA* (miR159d*, miR169m*, miR171h*/k*, miR319b*/d* and miR395o*) showed a greater number of normalized small RNA reads than the corresponding mature miRNA, and we even validated the potential targets of miR164f*, miR167g*/h*/i*, miR169r*, miR168a*/b* and miR827* by the degradome (Table 3), in line with previous findings from vertebrate [46] and *Medicago truncatula* [47]. These results suggested that these miRNAs* might be the authentic miRNAs, or both these miRNAs and corresponding miRNAs* could play important roles in gene expression regulation.

**Figure 6:**
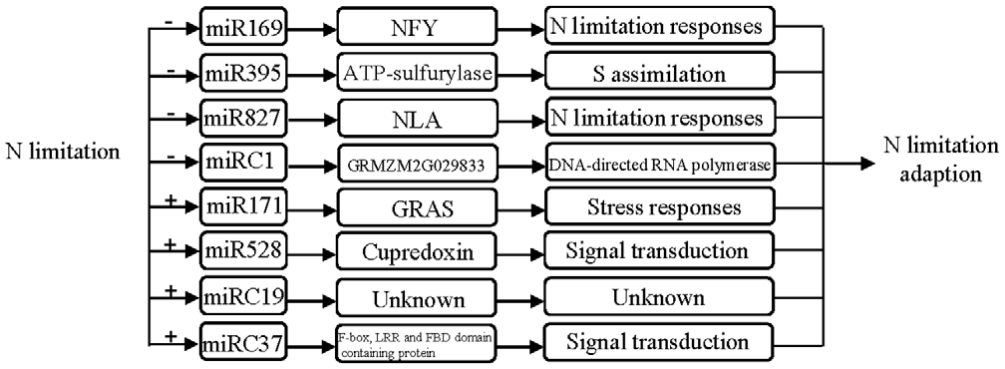
A possible functional network of N-limitation responsive miRNAs in maize seedlings. − represents negative regulation; + represents positive regulation.

We also discovered several miRNAs that were either downregulated or upregulated by N limitation, including the conserved miR169s, miR395s, miR528s, and miR827 and the newly identified miRC1, miR169w, miRC19, miRC37 and miR171p/q. As illustrated in Figure 6, the expression of miR169, miR395, miR827 and miRC1 was down-regulated by N-limitation and formed the first subgroup. In *Medicago truncatula*, miR169 is the key regulator for the differentiation of nodule primoridia [15]. Our previous research also showed that miR169 affected drought-resistance and N-limitation responses in *Arabidopsis* via inhibition of its targets, HAP2 transcription factors [17], [24]. Contrary to the low abundance of newly identified miRNAs, zm-miR169w was much more abundant than the conserved zma-miR169, indicating the important roles of zma-miR169w in various developmental processes or responses to abiotic stresses as is the case for other orthologs. miR827 is another miRNA that attracted our attention. The target genes of miR827 encoded SPX domain-containing proteins. One of its targets, *NITROGEN LIMITATION ADAPTATION* (NLA), is involved in adaptive responses to low N conditions in *Arabidopsis* [48]. Though recent evidence showed that miR827 took part in maintaining nitrate-dependent phosphate homeostasis in *Arabidopsis* [49], the function of miR827 in plant adaption to N limitation requires further research. miR395 was reported to function in regulating sulfur assimilation and translocation [50], [51], and the downregulation of miR395 indicated cross talk among different nutrient-deficient responses.

The second group consisted of 4 N-limitation downregulated miRNAs including miR171, miR528, miRC19 and miRC37. miR171 was predicted to GRAS family transcription factor-containing protein and the plant-specific GRAS gene family is essential for the activation of stress-inducible promoters [52]. The predicted targets of miR528 and miRC19 were involved signal transduction pathways. Recently, *NLP7* (nodule inception-like protein) was reported to be a positive regulator of the primary nitrate response [53]; *CHL1* (Nitrate Transporter *AtNRT1.1*) was assumed to utilize dual-affinity binding and a phosphorylation switch to sense a wide range of nitrate concentrations in the soil [54]; *NRT2.1*, a high-affinity nitrate transporter, might be the dominant nitrate sensor under nitrogen limited conditions [55]. However, a detailed understanding of the molecular basis underlying N sensing/signaling is still lacking. miR528 and miRC19 might shed another light on understanding the molecular basis underlying N sensing/signaling.

## Materials and Methods

### Plant materials and growth conditions

Plants of inbred *Zea mays* B73 were grown hydroponically in a growth chamber with a light intensity of 300 µmol·m^−2^·s^−1^, 60–70% relative humidity, and 28/22°C day/night temperature regime. Seeds of uniform size were surface sterilized in 10% H_2_O_2_ for 20 min and then germinated in coarse quartz sand at room temperature. The seedlings were then transferred to 2-L pots supplied with modified half-strength Hoagland's nutrient solution for 3 days and then supplied with full-strength Hoagland's nutrient solution. The basic nutrients in hydroponic solutions consisted of 0.75 mM K_2_SO_4_, 0.65 mM MgSO_4_·7H_2_O, 0.25 mM KH_2_PO_4_, 0.1 mM KCl, 2 mM Ca(NO_3_)_2_·4H_2_O, 0.1 mM Fe-EDTA, 0.01 mM H_3_BO_3_, 1 µM MnSO_4_·H_2_O, 1 µM ZnSO_4_·7H_2_O, 0.5 µM CuSO_4_·5H_2_O, and 0.005 µM (NH_4_)_6_Mo_7_O_24_·4H_2_O. The nutrient solution was replaced with fresh solution every 2 days. After they had grown for 2 weeks, seedlings were supplied with N-free nutrient solutions for 2 days to generate N-deficiency conditions. Nutrient solutions were renewed daily to ensure pH stability. Each treatment was replicated for three times. Shoots and roots were harvested separately, flash frozen and stored at −80°C.

### Small RNA isolation, library construction and sequencing

Total RNA was extracted from shoots and roots with TRIZOL reagent (Invitrogen). For enrichment of small RNAs, high molecular weight RNA was selectively precipitated by the addition of one volume of 20% PEG/1M NaCl [56]. The miRNAs were cloned as described by Sunkar & Zhu [16] with some modifications. In brief, low molecular weight RNA was fractioned on 17% denaturing polyacrylamide gels. Small RNAs in the range of 16–30 nt were excised and eluted with 0.3 M NaCl. The RNA was dephosphorylated and ligated sequentially to 5′ and 3′ RNA/DNA chimeric oligonucletide adaptors. Reverse transcription PCR was carried out and the resulting PCR products were sequenced using Solexa sequencing technology (LC Sciences, Hangzhou, China).

### Analysis of sequencing data

The raw reads were produced after excluding low quantity reads, and 5′ and 3′ adaptor contaminants. The identical adaptor-trimmed sequences in the range of 16–30 nt were grouped as unique sequences with normalized counts for the individual sequence reads. The unique small RNAs were aligned to the maize B73 RefGen_V2 genome (http://archive.maizesequence.org/index.html). Only perfectly matching sequences were considered for further analysis. Known non-coding RNAs derived from rRNAs, tRNAs, snRNAs and snoRNAs were identified by the National Center for Biotechnology Information (NCBI) BLASTN. The resulting small RNAs were mapped to the reported miRNAs from miRBase (miRBase15.0, http://www.mirbase.org/). These sequences were then clustered to generate the listed miRNA families. A unique sequence was identified as a new miRNA if it met the following criteria: the fold-back structures formed hairpins as predicted using RNAFOLD program [57]; the sequence was located in the duplex region of the hairpin structure [58]; and the corresponding miRNA* could be detected in the small RNA libraries [37]. For prediction of miRNA target genes, the procedure and criteria were followed as described by Allen et al. [41] and Schwab et al. [42].

### RNA analysis

Low molecular weight RNA was separated from total RNA by PEG precipitation. Small RNA (40 µg per lane) was loaded and the blots were probed and washed as previously reported [17]. The probes used were listed in Table S7.

For real-time RT-PCR, 5 µg of total RNA isolated with the RNeasy plant mini kit was used for the first-strand cDNA synthesis using SuperScript III first-strand synthesis supermix (Invitrogen). The cDNA reaction mixture was diluted three times, and 1 µL was used as template in a 25-µL PCR reaction. PCR was performed after a preincubation at 95°C for 3 min and was followed by 40 cycles of denaturation at 95°C for 15 s, annealing at 60°C for 40 s, and extension at 72°C for 40 s. All the reactions were performed in the iQ5 real-time PCR detection system using iQ SYBR green supermix (Bio-Rad). Primers specific for *MIR169C/R* were used to detect expression levels of miR169c/r. Each experiment was replicated three times. The comparative Ct method was applied.

### 5′ RACE

To obtain cleavage fragments resulting from transcript processing by microRNAs, 5′ RACE System for Rapid Amplification of cDNA Ends, Version 2.0 (Invitrogen) was used. Total RNA was extracted from N-deficient roots by the TRIZOL method described above. The oligo dT was then used for cDNA synthesis. Initial PCR was carried out using the 5′ RACE Abridged Primer and gene specific outer primer. Nested PCR was carried out using the 1/100 of the initial PCR reaction, Universal Amplification Primer and gene specific inner primer. The gene specific primers were listed in Table S7.

### Degradome library construction and data analysis

A degradome library was constructed for hydroponically grown 2-week-old maize roots that had been subjected to N stress for 2 d as described by German et al. [39] and Addo-Quaye et al. [40] with some modifications. In brief, 1 mg of polyA-enriched RNA was ligated to a 5′ RNA adaptor with a Mmel site, followed by reverse transcription PCR (five cycles), Mmel digestion, PAGE-gel purification, ligation of 3′-double strand DNA adaptor, PAGE-gel purification, PCR amplication (21 cycles), and PAGE-gel purification. The purified cDNA library was sequenced with a Solaxa/Illumina genome analyzer (LC Sciences, Hangzhou, China). A public software package, CleaveLand, was used for analyzing sequencing data Addo-Quaye et al. [59], [60].

## Supporting Information

Table S1 Summary statistics of small RNA sequences from maize shoots and roots with/without N.
(PPT)

Table S2 Different categories of small RNAs by deep sequencing.
(PPT)

Table S3 Sequences of novel miRNAs identified by small RNA deep sequencing in maize.
(PPT)

Table S4 Expression profiles of new miRNAs identified by small RNA library sequencing and degradome sequencing.
(PPT)

Table S5 Normalized abundance of known-miRNAs in response to N deficiency.
(XLS)

Table S6 Predicted targets of novel miRNAs identified by small RNA deep sequencing in maize.
(PPT)

Table S7 Probes and primers used in this study.
(PPT)
