# Supplementary material for: Cloning and Characterization of Maize miRNAs Involved in Responses to Nitrogen Deficiency
Source: PLoS One. 2012 Jan 3;7(1):e29669. doi: 10.1371/journal.pone.0029669 (PMC3250470; doi:10.1371/journal.pone.0029669)
Supplement: Table S1 — Summary statistics of small RNA sequences from maize shoots and roots with/without N. (PPT) [file pone.0029669.s001.ppt]

## Slide 1
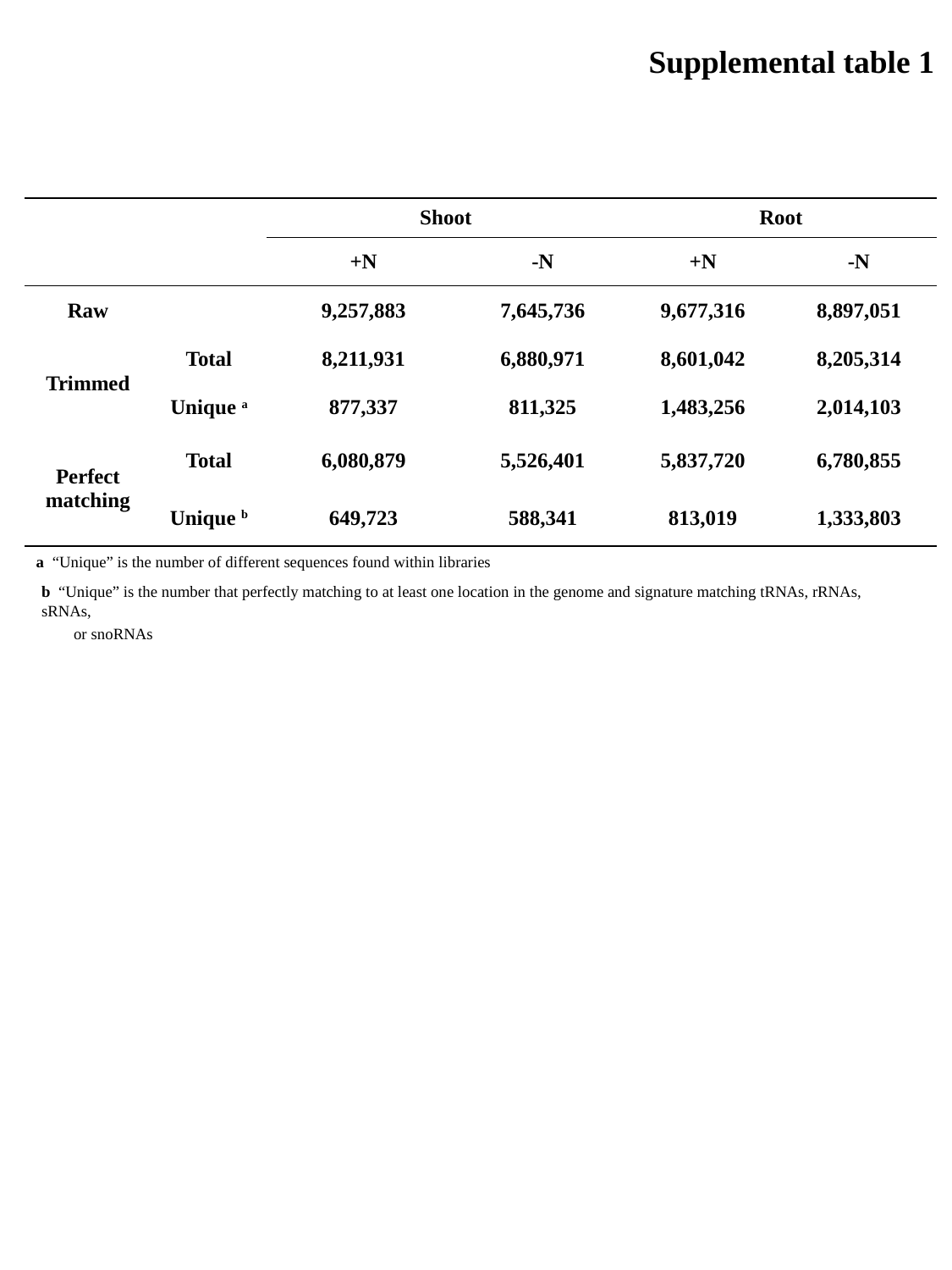

Supplemental table 1
| | | Shoot | | Root | |
| --- | --- | --- | --- | --- | --- |
| | | +N | -N | +N | -N |
| Raw | | 9,257,883 | 7,645,736 | 9,677,316 | 8,897,051 |
| Trimmed | Total | 8,211,931 | 6,880,971 | 8,601,042 | 8,205,314 |
| | Unique a | 877,337 | 811,325 | 1,483,256 | 2,014,103 |
| Perfect matching | Total | 6,080,879 | 5,526,401 | 5,837,720 | 6,780,855 |
| | Unique b | 649,723 | 588,341 | 813,019 | 1,333,803 |
a “Unique” is the number of different sequences found within libraries
b “Unique” is the number that perfectly matching to at least one location in the genome and signature matching tRNAs, rRNAs, sRNAs,
 or snoRNAs
