# Supplementary material for: Cloning and Characterization of Maize miRNAs Involved in Responses to Nitrogen Deficiency
Source: PLoS One. 2012 Jan 3;7(1):e29669. doi: 10.1371/journal.pone.0029669 (PMC3250470; doi:10.1371/journal.pone.0029669)
Supplement: Table S2 — Different categories of small RNAs by deep sequencing. (PPT) [file pone.0029669.s002.ppt]

## Slide 1
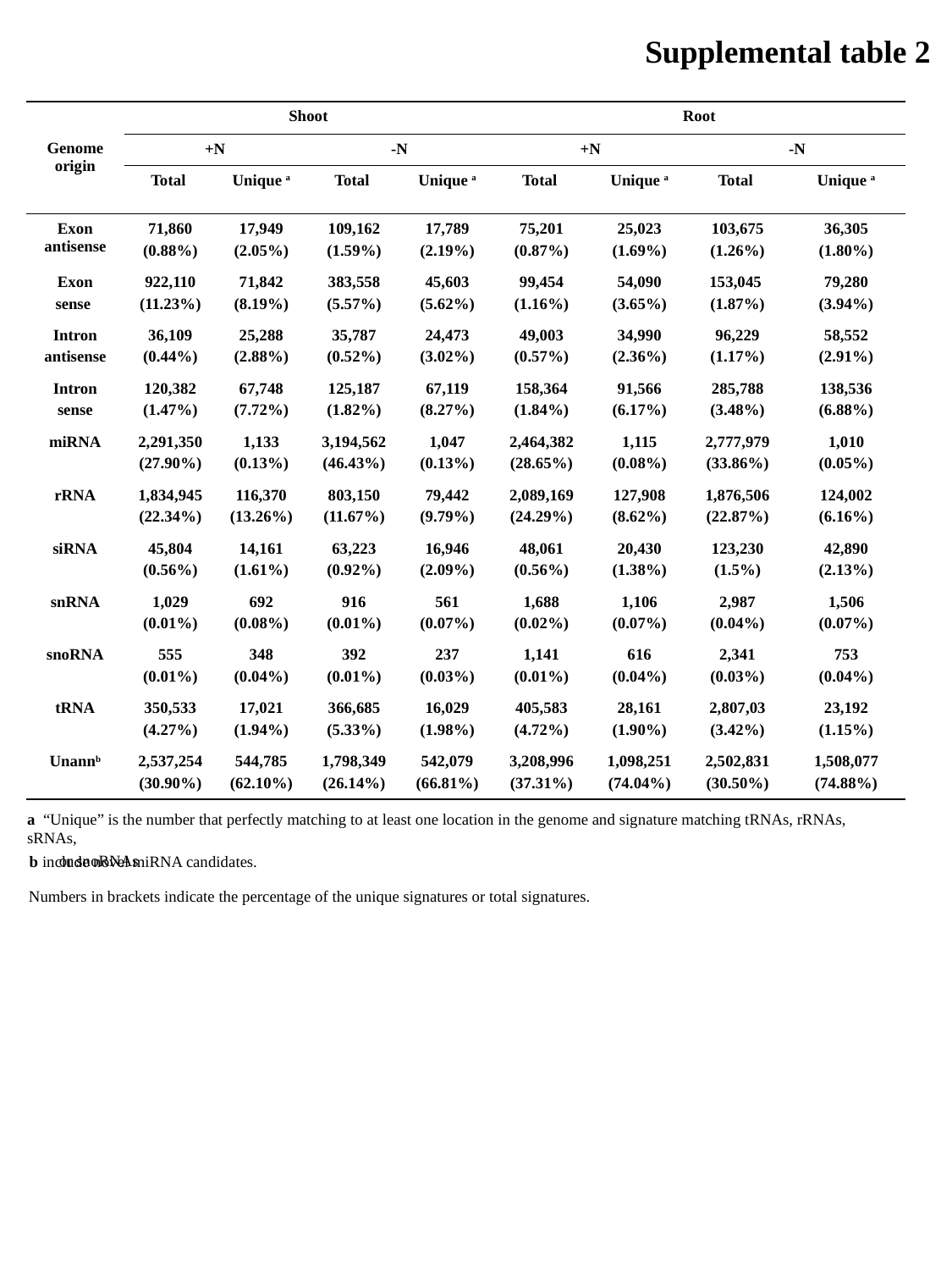

Supplemental table 2
| Genome origin | Shoot | | | | Root | | | |
| --- | --- | --- | --- | --- | --- | --- | --- | --- |
| | +N | | -N | | +N | | -N | |
| | Total | Unique a | Total | Unique a | Total | Unique a | Total | Unique a |
| Exon antisense | 71,860 (0.88%) | 17,949 (2.05%) | 109,162 (1.59%) | 17,789 (2.19%) | 75,201 (0.87%) | 25,023 (1.69%) | 103,675 (1.26%) | 36,305 (1.80%) |
| Exon sense | 922,110 (11.23%) | 71,842 (8.19%) | 383,558 (5.57%) | 45,603 (5.62%) | 99,454 (1.16%) | 54,090 (3.65%) | 153,045 (1.87%) | 79,280 (3.94%) |
| Intron antisense | 36,109 (0.44%) | 25,288 (2.88%) | 35,787 (0.52%) | 24,473 (3.02%) | 49,003 (0.57%) | 34,990 (2.36%) | 96,229 (1.17%) | 58,552 (2.91%) |
| Intron sense | 120,382 (1.47%) | 67,748 (7.72%) | 125,187 (1.82%) | 67,119 (8.27%) | 158,364 (1.84%) | 91,566 (6.17%) | 285,788 (3.48%) | 138,536 (6.88%) |
| miRNA | 2,291,350 (27.90%) | 1,133 (0.13%) | 3,194,562 (46.43%) | 1,047 (0.13%) | 2,464,382 (28.65%) | 1,115 (0.08%) | 2,777,979 (33.86%) | 1,010 (0.05%) |
| rRNA | 1,834,945 (22.34%) | 116,370 (13.26%) | 803,150 (11.67%) | 79,442 (9.79%) | 2,089,169 (24.29%) | 127,908 (8.62%) | 1,876,506 (22.87%) | 124,002 (6.16%) |
| siRNA | 45,804 (0.56%) | 14,161 (1.61%) | 63,223 (0.92%) | 16,946 (2.09%) | 48,061 (0.56%) | 20,430 (1.38%) | 123,230 (1.5%) | 42,890 (2.13%) |
| snRNA | 1,029 (0.01%) | 692 (0.08%) | 916 (0.01%) | 561 (0.07%) | 1,688 (0.02%) | 1,106 (0.07%) | 2,987 (0.04%) | 1,506 (0.07%) |
| snoRNA | 555 (0.01%) | 348 (0.04%) | 392 (0.01%) | 237 (0.03%) | 1,141 (0.01%) | 616 (0.04%) | 2,341 (0.03%) | 753 (0.04%) |
| tRNA | 350,533 (4.27%) | 17,021 (1.94%) | 366,685 (5.33%) | 16,029 (1.98%) | 405,583 (4.72%) | 28,161 (1.90%) | 2,807,03 (3.42%) | 23,192 (1.15%) |
| Unannb | 2,537,254 (30.90%) | 544,785 (62.10%) | 1,798,349 (26.14%) | 542,079 (66.81%) | 3,208,996 (37.31%) | 1,098,251 (74.04%) | 2,502,831 (30.50%) | 1,508,077 (74.88%) |
a “Unique” is the number that perfectly matching to at least one location in the genome and signature matching tRNAs, rRNAs, sRNAs,
 or snoRNAs
b include novel miRNA candidates.
Numbers in brackets indicate the percentage of the unique signatures or total signatures.
