# Supplementary material for: Cloning and Characterization of Maize miRNAs Involved in Responses to Nitrogen Deficiency
Source: PLoS One. 2012 Jan 3;7(1):e29669. doi: 10.1371/journal.pone.0029669 (PMC3250470; doi:10.1371/journal.pone.0029669)
Supplement: Table S3 — Sequences of novel miRNAs identified by small RNA deep sequencing in maize. (PPT) [file pone.0029669.s003.ppt]

## Slide 1
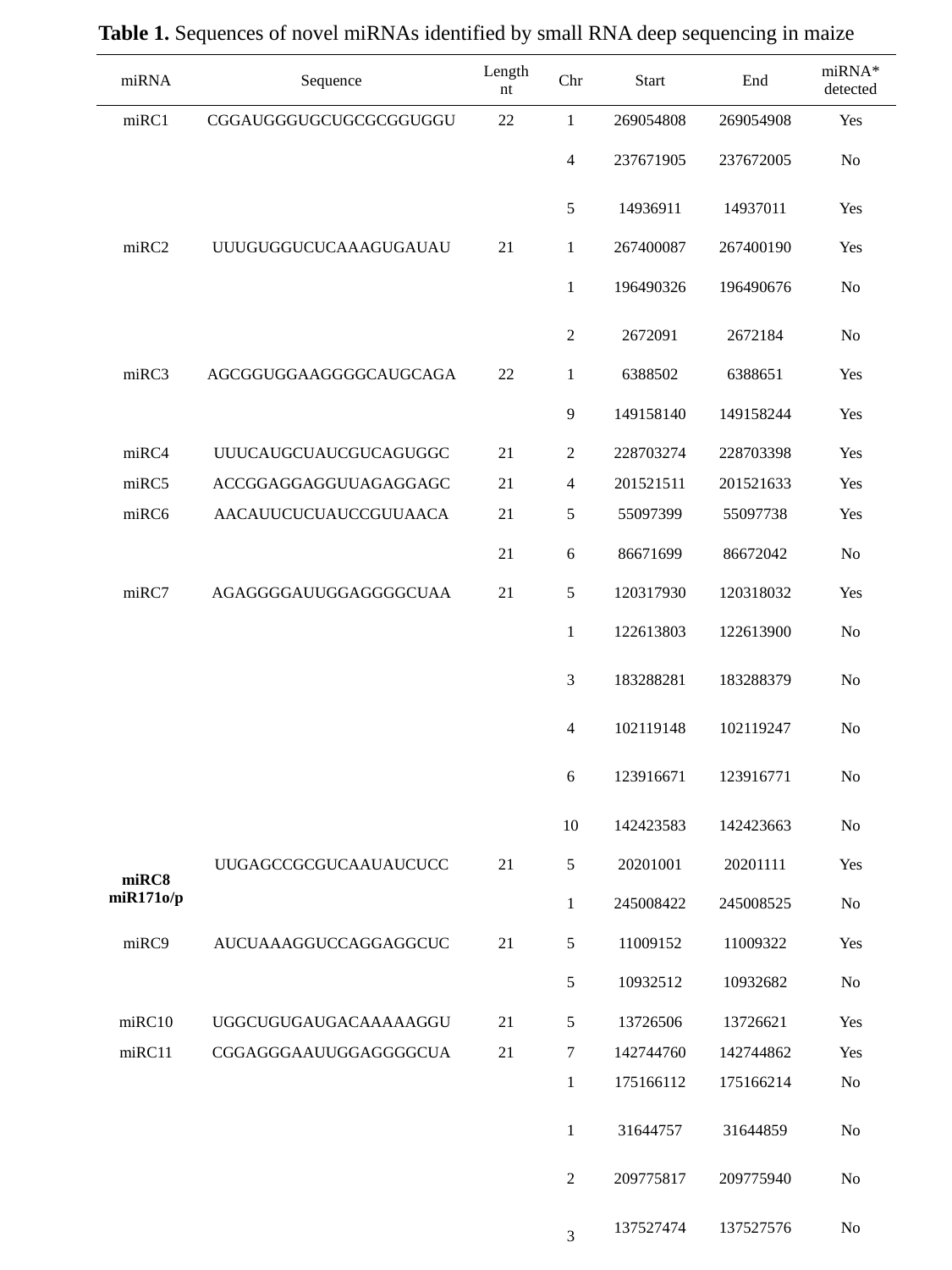

| | | | | | | | | |
| --- | --- | --- | --- | --- | --- | --- | --- | --- |
| | | | | | | | | |
| | | | | | | | | |
| | | | | | | | | |
| | | | | | | | | |
| | | | | | | | | |
| | | | | | | | | |
| | | | | | | | | |
Table 1. Sequences of novel miRNAs identified by small RNA deep sequencing in maize
| miRNA | Sequence | Length nt | Chr | Start | End | miRNA\* detected |
| --- | --- | --- | --- | --- | --- | --- |
| miRC1 | CGGAUGGGUGCUGCGCGGUGGU | 22 | 1 | 269054808 | 269054908 | Yes |
| | | | 4 | 237671905 | 237672005 | No |
| | | | 5 | 14936911 | 14937011 | Yes |
| miRC2 | UUUGUGGUCUCAAAGUGAUAU | 21 | 1 | 267400087 | 267400190 | Yes |
| | | | 1 | 196490326 | 196490676 | No |
| | | | 2 | 2672091 | 2672184 | No |
| miRC3 | AGCGGUGGAAGGGGCAUGCAGA | 22 | 1 | 6388502 | 6388651 | Yes |
| | | | 9 | 149158140 | 149158244 | Yes |
| miRC4 | UUUCAUGCUAUCGUCAGUGGC | 21 | 2 | 228703274 | 228703398 | Yes |
| miRC5 | ACCGGAGGAGGUUAGAGGAGC | 21 | 4 | 201521511 | 201521633 | Yes |
| miRC6 | AACAUUCUCUAUCCGUUAACA | 21 | 5 | 55097399 | 55097738 | Yes |
| | | 21 | 6 | 86671699 | 86672042 | No |
| miRC7 | AGAGGGGAUUGGAGGGGCUAA | 21 | 5 | 120317930 | 120318032 | Yes |
| | | | 1 | 122613803 | 122613900 | No |
| | | | 3 | 183288281 | 183288379 | No |
| | | | 4 | 102119148 | 102119247 | No |
| | | | 6 | 123916671 | 123916771 | No |
| | | | 10 | 142423583 | 142423663 | No |
| miRC8 miR171o/p | UUGAGCCGCGUCAAUAUCUCC | 21 | 5 | 20201001 | 20201111 | Yes |
| | | | 1 | 245008422 | 245008525 | No |
| miRC9 | AUCUAAAGGUCCAGGAGGCUC | 21 | 5 | 11009152 | 11009322 | Yes |
| | | | 5 | 10932512 | 10932682 | No |
| miRC10 | UGGCUGUGAUGACAAAAAGGU | 21 | 5 | 13726506 | 13726621 | Yes |
| miRC11 | CGGAGGGAAUUGGAGGGGCUA | 21 | 7 | 142744760 | 142744862 | Yes |
| | | | 1 | 175166112 | 175166214 | No |
| | | | 1 | 31644757 | 31644859 | No |
| | | | 2 | 209775817 | 209775940 | No |
| | | | 3 | 137527474 | 137527576 | No |
| | | | 5 | 162713223 | 162713325 | No |
| | | | 5 | 203138724 | 203138825 | No |
| miRC12 | AUGGUGCAUUGACUUGGUCAA | 21 | 8 | 101968490 | 101968619 | Yes |
| miRC13 | UUAGGCUCGGGGACUACGGUG | 21 | 8 | 4766569 | 4766746 | Yes |
| miRC14 | UUCUCCAGGAGUUGAUGGACAA | 22 | 8 | 39157879 | 39158234 | Yes |
| miRC15 | UGAAAAGCUAGAACGAUUUAC | 21 | 9 | 33858898 | 33859002 | Yes |
| miRC16 miR169s | UAGCCAAGCAUGAUUUGCCCG | 21 | 1 | 297173995 | 297174083 | Yes |
| miRC17 | GGAUCACAGGAGGAUUGGAGG | 21 | 10 | 144082489 | 144082588 | Yes |
| | | | 1 | 172912423 | 172912521 | No |
| | | | 1 | 229953458 | 229953556 | No |
| | | | 1 | 35925993 | 35926092 | No |
| | | | 2 | 46971263 | 46971362 | No |
| | | | 5 | 165910017 | 165910118 | No |
| | | | 10 | 75870181 | 75870280 | No |
| miRC18 | AUGGAGUGGAUUGAGGGGGCU | 21 | 1 | 290825588 | 290825690 | Yes |
| | | | 1 | 90262274 | 90262376 | No |
| | | | 2 | 209680336 | 209680438 | No |
| | | | 5 | 7571049 | 7571150 | No |
| miRC19 | AUGCGGAGAGGCUCUCGAGAGA | 22 | 5 | 91651975 | 91652130 | Yes |
| miRC20 | AGACUUAGGAACGGAGGGAGU | 21 | 2 | 162419627 | 162419952 | Yes |
| miRC21 | UGUGGAUUAGGUGGGAUUGGA | 21 | 2 | 161834278 | 161834382 | Yes |
| | | | 3 | 124135955 | 124136058 | No |
| | | | 5 | 170572936 | 170573044 | No |
| | | | 6 | 112428620 | 112428724 | No |
| | | | 8 | 148426150 | 148426254 | No |
| miRC22 | UUAUCUCCGACGGCUAGUUAC | 21 | 1 | 19016981 | 19017079 | Yes |
| | | | 5 | 106000150 | 106000268 | No |
| miRC23 | GAUCCCCAGCGGAGUCGCCA | 20 | 1 | 275868775 | 275869078 | Yes |
| miRC24 miR398c | CGGCGGGGGCGAACUGAGAAC | 21 | 2 | 166626792 | 166626958 | Yes |
| miRC25 | GAGGGGAUUGAAGGGGUUAGA | 21 | 2 | 14466264 | 14466360 | Yes |

## Slide 2
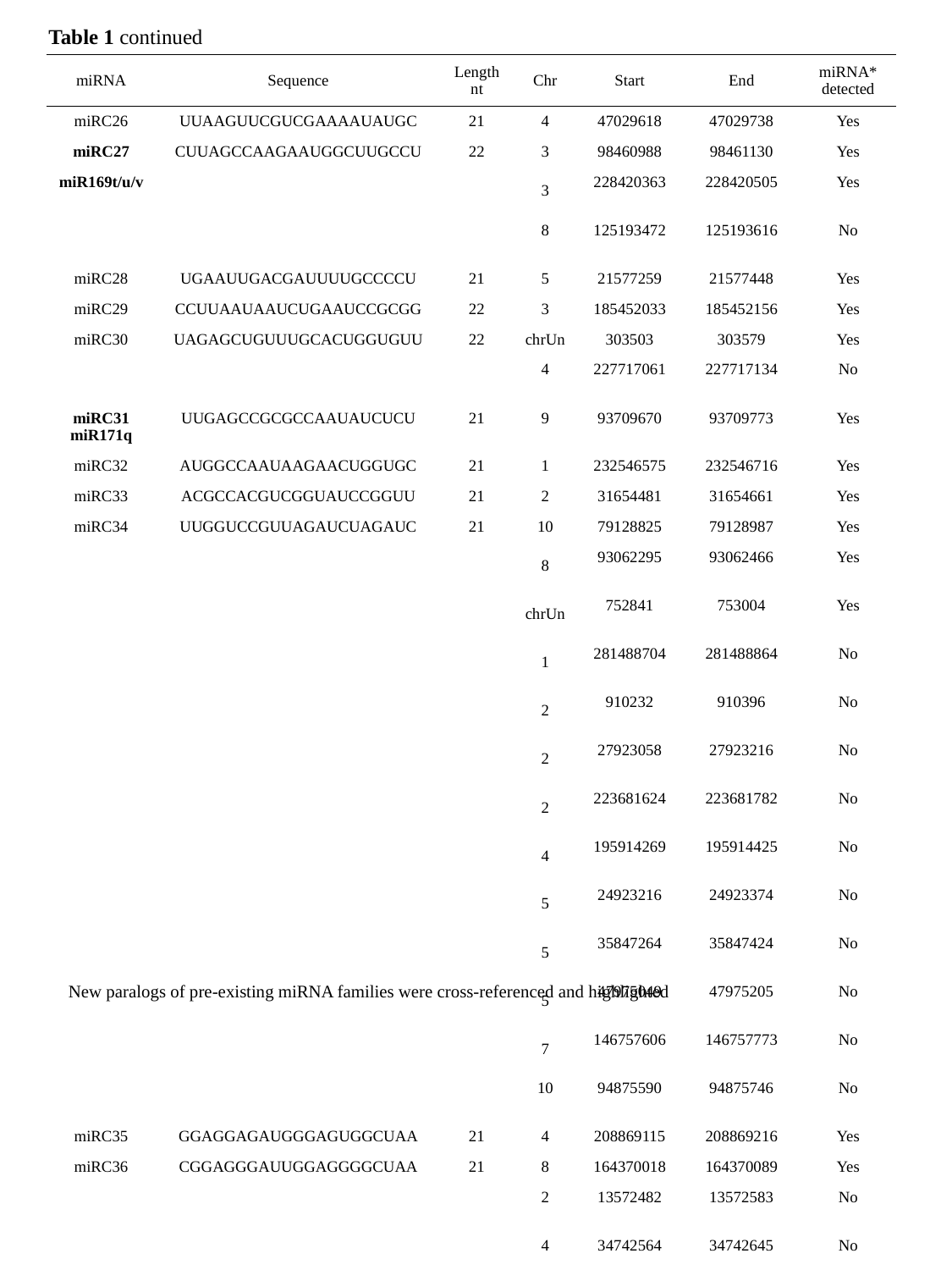

Table 1 continued
| miRNA | Sequence | Length nt | Chr | Start | End | miRNA\* detected |
| --- | --- | --- | --- | --- | --- | --- |
| miRC26 | UUAAGUUCGUCGAAAAUAUGC | 21 | 4 | 47029618 | 47029738 | Yes |
| miRC27 | CUUAGCCAAGAAUGGCUUGCCU | 22 | 3 | 98460988 | 98461130 | Yes |
| miR169t/u/v | | | 3 | 228420363 | 228420505 | Yes |
| | | | 8 | 125193472 | 125193616 | No |
| miRC28 | UGAAUUGACGAUUUUGCCCCU | 21 | 5 | 21577259 | 21577448 | Yes |
| miRC29 | CCUUAAUAAUCUGAAUCCGCGG | 22 | 3 | 185452033 | 185452156 | Yes |
| miRC30 | UAGAGCUGUUUGCACUGGUGUU | 22 | chrUn | 303503 | 303579 | Yes |
| | | | 4 | 227717061 | 227717134 | No |
| miRC31 miR171q | UUGAGCCGCGCCAAUAUCUCU | 21 | 9 | 93709670 | 93709773 | Yes |
| miRC32 | AUGGCCAAUAAGAACUGGUGC | 21 | 1 | 232546575 | 232546716 | Yes |
| miRC33 | ACGCCACGUCGGUAUCCGGUU | 21 | 2 | 31654481 | 31654661 | Yes |
| miRC34 | UUGGUCCGUUAGAUCUAGAUC | 21 | 10 | 79128825 | 79128987 | Yes |
| | | | 8 | 93062295 | 93062466 | Yes |
| | | | chrUn | 752841 | 753004 | Yes |
| | | | 1 | 281488704 | 281488864 | No |
| | | | 2 | 910232 | 910396 | No |
| | | | 2 | 27923058 | 27923216 | No |
| | | | 2 | 223681624 | 223681782 | No |
| | | | 4 | 195914269 | 195914425 | No |
| | | | 5 | 24923216 | 24923374 | No |
| | | | 5 | 35847264 | 35847424 | No |
| | | | 5 | 47975049 | 47975205 | No |
| | | | 7 | 146757606 | 146757773 | No |
| | | | 10 | 94875590 | 94875746 | No |
| miRC35 | GGAGGAGAUGGGAGUGGCUAA | 21 | 4 | 208869115 | 208869216 | Yes |
| miRC36 | CGGAGGGAUUGGAGGGGCUAA | 21 | 8 | 164370018 | 164370089 | Yes |
| | | | 2 | 13572482 | 13572583 | No |
| | | | 4 | 34742564 | 34742645 | No |
| | | | 8 | 58479917 | 58480040 | No |
| miRC37 | CCGGAGGGGAUUGGAGGGGCU | 21 | 7 | 110577552 | 110577661 | Yes |
| miRC38 | UAGUCCCUAAAUUGCCGGACA | 21 | 10 | 130456852 | 130456951 | Yes |
| miRC39 | CCUGGGCGGCAGGGCGAUGGCU | 22 | 2 | 57342716 | 57342951 | Yes |
| miRC40 | CAGCACCUGCAUACUUUGCUUC | 22 | 4 | 19910089 | 19910184 | Yes |
| miRC41 | AUGGGCUUUAGCAGUUAACUAGA | 23 | 8 | 2066988 | 2067085 | Yes |
| miRC42 | CGGAGGGGAUUGGAGAGGCUA | 21 | 2 | 6138947 | 6139049 | Yes |
| | | | 1 | 232558390 | 232558487 | No |
| | | | 6 | 145814514 | 145814616 | No |
| | | | 8 | 171785981 | 171786087 | No |
| miRC43 | AAUACACAUGGGUUGAGGGAG | 21 | 5 | 140705141 | 140705237 | Yes |
| | | | 2 | 134442204 | 134442300 | No |
| miRC44 | CGAGAGGCUGUCAUAUCGACC | 21 | 5 | 71871206 | 71871308 | Yes |
| | | | 5 | 71849434 | 71849536 | No |
| miRC45 | UAUGAACCUUGAUUAGCAACAU | 22 | 7 | 132286498 | 132286761 | Yes |
| miRC46 | AUCGUGGGGAUUACAUGCGGA | 21 | 9 | 119104729 | 119104889 | Yes |
New paralogs of pre-existing miRNA families were cross-referenced and highlighted
