# Supplementary material for: Cloning and Characterization of Maize miRNAs Involved in Responses to Nitrogen Deficiency
Source: PLoS One. 2012 Jan 3;7(1):e29669. doi: 10.1371/journal.pone.0029669 (PMC3250470; doi:10.1371/journal.pone.0029669)
Supplement: Table S4 — Expression profiles of new miRNAs identified by small RNA library sequencing and degradome sequencing. (PPT) [file pone.0029669.s004.ppt]

## Slide 1
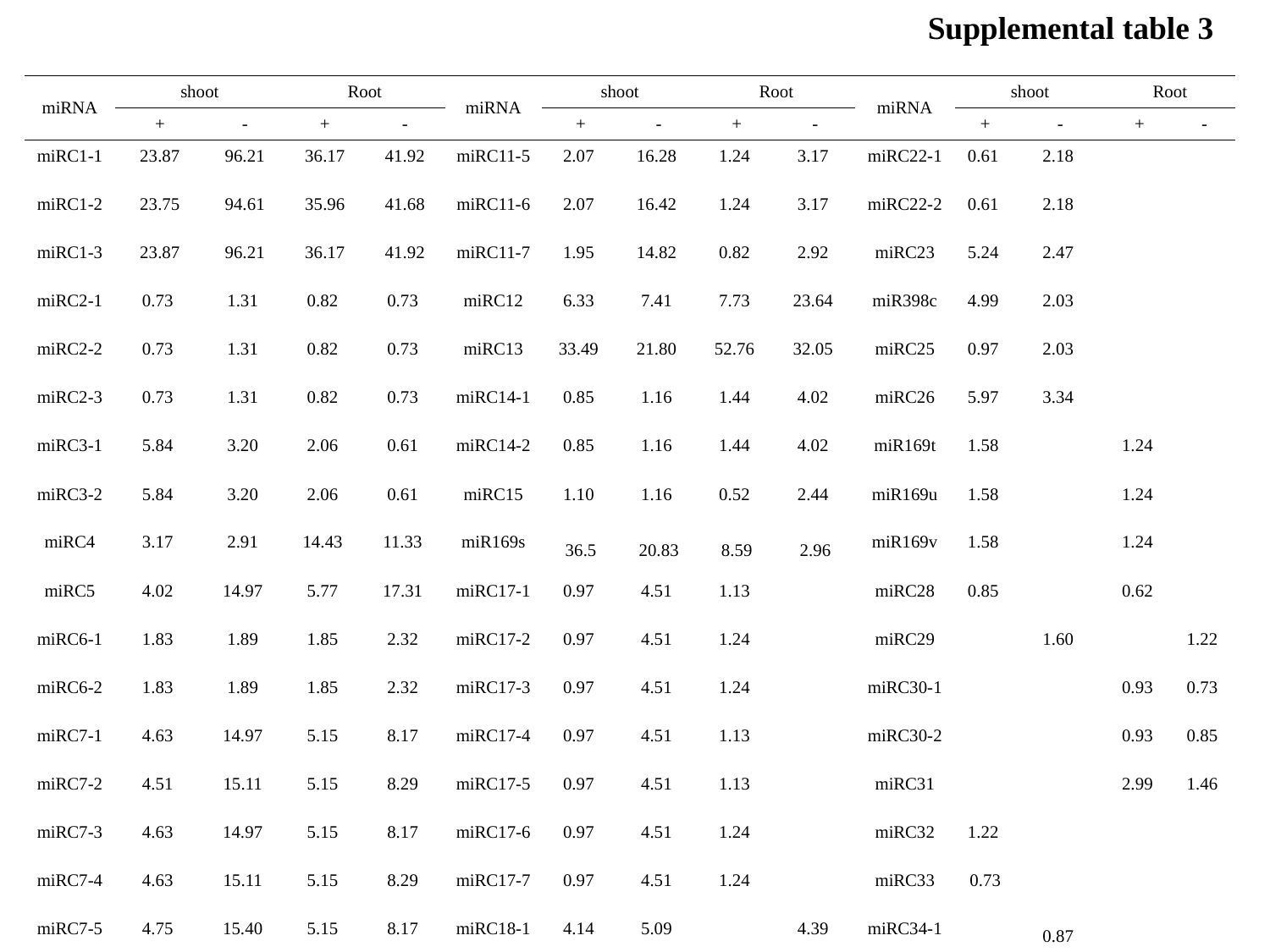

Supplemental table 3
| miRNA | shoot | | Root | | miRNA | shoot | | Root | | miRNA | shoot | | Root | |
| --- | --- | --- | --- | --- | --- | --- | --- | --- | --- | --- | --- | --- | --- | --- |
| | + | - | + | - | | + | - | + | - | | + | - | + | - |
| miRC1-1 | 23.87 | 96.21 | 36.17 | 41.92 | miRC11-5 | 2.07 | 16.28 | 1.24 | 3.17 | miRC22-1 | 0.61 | 2.18 | | |
| miRC1-2 | 23.75 | 94.61 | 35.96 | 41.68 | miRC11-6 | 2.07 | 16.42 | 1.24 | 3.17 | miRC22-2 | 0.61 | 2.18 | | |
| miRC1-3 | 23.87 | 96.21 | 36.17 | 41.92 | miRC11-7 | 1.95 | 14.82 | 0.82 | 2.92 | miRC23 | 5.24 | 2.47 | | |
| miRC2-1 | 0.73 | 1.31 | 0.82 | 0.73 | miRC12 | 6.33 | 7.41 | 7.73 | 23.64 | miR398c | 4.99 | 2.03 | | |
| miRC2-2 | 0.73 | 1.31 | 0.82 | 0.73 | miRC13 | 33.49 | 21.80 | 52.76 | 32.05 | miRC25 | 0.97 | 2.03 | | |
| miRC2-3 | 0.73 | 1.31 | 0.82 | 0.73 | miRC14-1 | 0.85 | 1.16 | 1.44 | 4.02 | miRC26 | 5.97 | 3.34 | | |
| miRC3-1 | 5.84 | 3.20 | 2.06 | 0.61 | miRC14-2 | 0.85 | 1.16 | 1.44 | 4.02 | miR169t | 1.58 | | 1.24 | |
| miRC3-2 | 5.84 | 3.20 | 2.06 | 0.61 | miRC15 | 1.10 | 1.16 | 0.52 | 2.44 | miR169u | 1.58 | | 1.24 | |
| miRC4 | 3.17 | 2.91 | 14.43 | 11.33 | miR169s | 36.5 | 20.83 | 8.59 | 2.96 | miR169v | 1.58 | | 1.24 | |
| miRC5 | 4.02 | 14.97 | 5.77 | 17.31 | miRC17-1 | 0.97 | 4.51 | 1.13 | | miRC28 | 0.85 | | 0.62 | |
| miRC6-1 | 1.83 | 1.89 | 1.85 | 2.32 | miRC17-2 | 0.97 | 4.51 | 1.24 | | miRC29 | | 1.60 | | 1.22 |
| miRC6-2 | 1.83 | 1.89 | 1.85 | 2.32 | miRC17-3 | 0.97 | 4.51 | 1.24 | | miRC30-1 | | | 0.93 | 0.73 |
| miRC7-1 | 4.63 | 14.97 | 5.15 | 8.17 | miRC17-4 | 0.97 | 4.51 | 1.13 | | miRC30-2 | | | 0.93 | 0.85 |
| miRC7-2 | 4.51 | 15.11 | 5.15 | 8.29 | miRC17-5 | 0.97 | 4.51 | 1.13 | | miRC31 | | | 2.99 | 1.46 |
| miRC7-3 | 4.63 | 14.97 | 5.15 | 8.17 | miRC17-6 | 0.97 | 4.51 | 1.24 | | miRC32 | 1.22 | | | |
| miRC7-4 | 4.63 | 15.11 | 5.15 | 8.29 | miRC17-7 | 0.97 | 4.51 | 1.24 | | miRC33 | 0.73 | | | |
| miRC7-5 | 4.75 | 15.40 | 5.15 | 8.17 | miRC18-1 | 4.14 | 5.09 | | 4.39 | miRC34-1 | | 0.87 | | |
| miRC7-6 | 4.63 | 15.26 | 5.15 | 8.17 | miRC18-2 | 3.77 | 4.51 | | 3.78 | miRC34-2 | | 0.87 | | |
| miR171o | 24.96 | 25.29 | 1.24 | 5.36 | miRC18-3 | 4.14 | 4.51 | | 3.78 | miRC34-3 | | 0.87 | | |
| miR171q | 24.96 | 25.29 | 1.24 | 5.36 | miRC18-4 | 4.14 | 5.09 | | 4.39 | miRC34-4 | | 0.87 | | |
| miRC9-1 | 0.73 | 1.16 | 0.62 | 1.71 | miRC19 | 1.95 | 8.86 | | 1.22 | miRC34-5 | | 0.87 | | |
| miRC9-2 | 0.73 | 1.16 | 0.62 | 1.71 | miRC20 | | 2.47 | 0.72 | 1.10 | miRC34-6 | | 0.87 | | |
| miRC10 | 0.97 | 1.16 | 1.65 | 3.29 | miRC21-1 | | 1.45 | 1.34 | 2.92 | miRC34-7 | | 0.87 | | |
| miRC11-1 | 2.07 | 16.28 | 1.13 | 3.17 | miRC21-2 | | 1.31 | 1.24 | 2.80 | miRC34-8 | | 0.87 | | |
| miRC11-2 | 2.07 | 16.28 | 1.24 | 3.17 | miRC21-3 | | 1.31 | 1.24 | 2.80 | miRC34-9 | | 0.87 | | |
| miRC11-3 | 2.07 | 16.28 | 1.24 | 3.17 | miRC21-4 | | 1.31 | 1.24 | 3.17 | miRC34-10 | | 0.87 | | |
| miRC11-4 | 2.07 | 16.42 | 1.24 | 3.17 | miRC21-5 | | 1.31 | 1.24 | 2.80 | miRC34-11 | | 0.87 | | |

## Slide 2
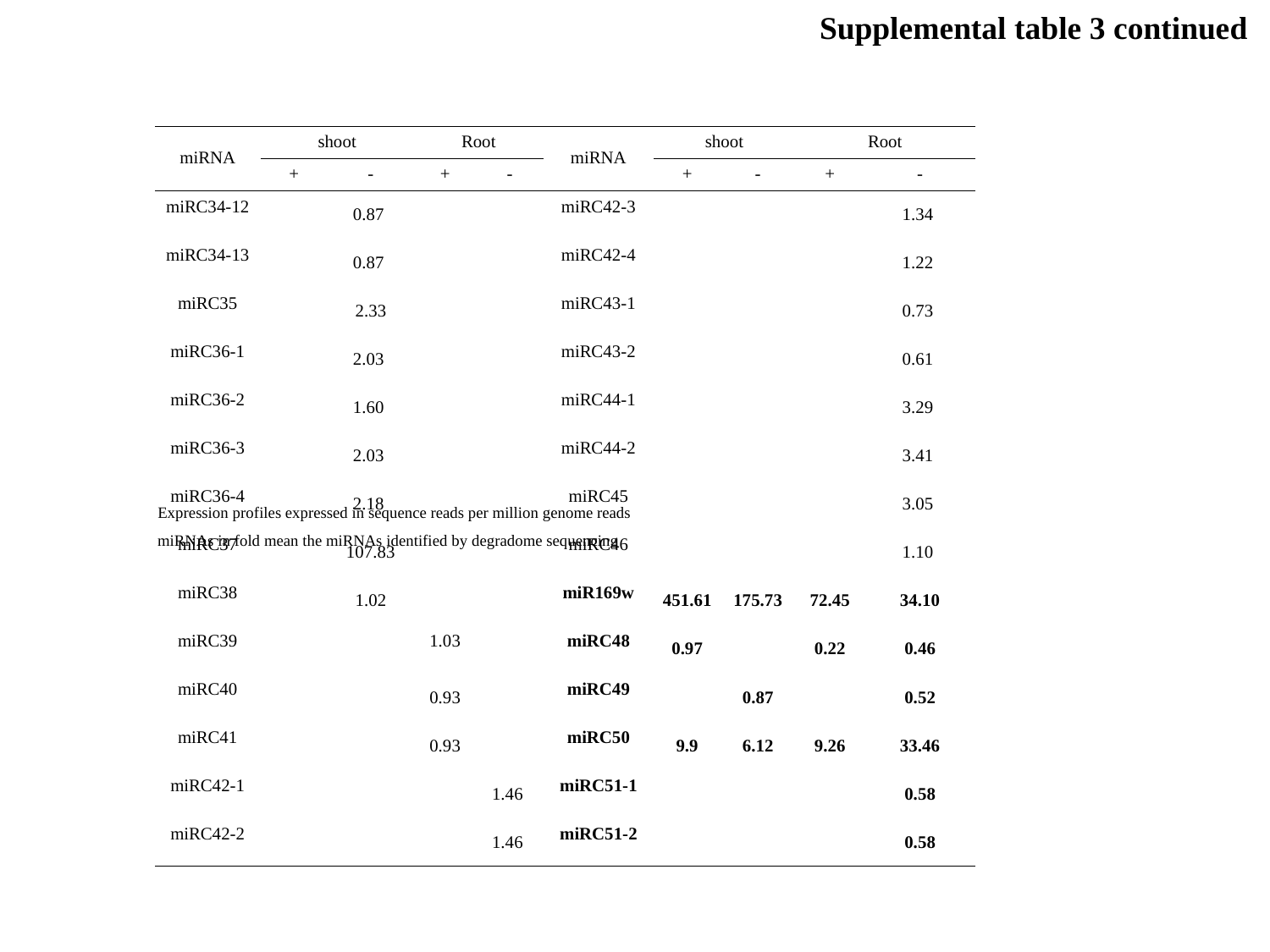

Supplemental table 3 continued
| miRNA | shoot | | Root | | miRNA | shoot | | Root | |
| --- | --- | --- | --- | --- | --- | --- | --- | --- | --- |
| | + | - | + | - | | + | - | + | - |
| miRC34-12 | | 0.87 | | | miRC42-3 | | | | 1.34 |
| miRC34-13 | | 0.87 | | | miRC42-4 | | | | 1.22 |
| miRC35 | | 2.33 | | | miRC43-1 | | | | 0.73 |
| miRC36-1 | | 2.03 | | | miRC43-2 | | | | 0.61 |
| miRC36-2 | | 1.60 | | | miRC44-1 | | | | 3.29 |
| miRC36-3 | | 2.03 | | | miRC44-2 | | | | 3.41 |
| miRC36-4 | | 2.18 | | | miRC45 | | | | 3.05 |
| miRC37 | | 107.83 | | | miRC46 | | | | 1.10 |
| miRC38 | | 1.02 | | | miR169w | 451.61 | 175.73 | 72.45 | 34.10 |
| miRC39 | | | 1.03 | | miRC48 | 0.97 | | 0.22 | 0.46 |
| miRC40 | | | 0.93 | | miRC49 | | 0.87 | | 0.52 |
| miRC41 | | | 0.93 | | miRC50 | 9.9 | 6.12 | 9.26 | 33.46 |
| miRC42-1 | | | | 1.46 | miRC51-1 | | | | 0.58 |
| miRC42-2 | | | | 1.46 | miRC51-2 | | | | 0.58 |
Expression profiles expressed in sequence reads per million genome reads
miRNAs in fold mean the miRNAs identified by degradome sequencing
