# Supplementary material for: Cloning and Characterization of Maize miRNAs Involved in Responses to Nitrogen Deficiency
Source: PLoS One. 2012 Jan 3;7(1):e29669. doi: 10.1371/journal.pone.0029669 (PMC3250470; doi:10.1371/journal.pone.0029669)
Supplement: Table S6 — Predicted targets of novel miRNAs identified by small RNA deep sequencing in maize. (PPT) [file pone.0029669.s006.ppt]

## Slide 1
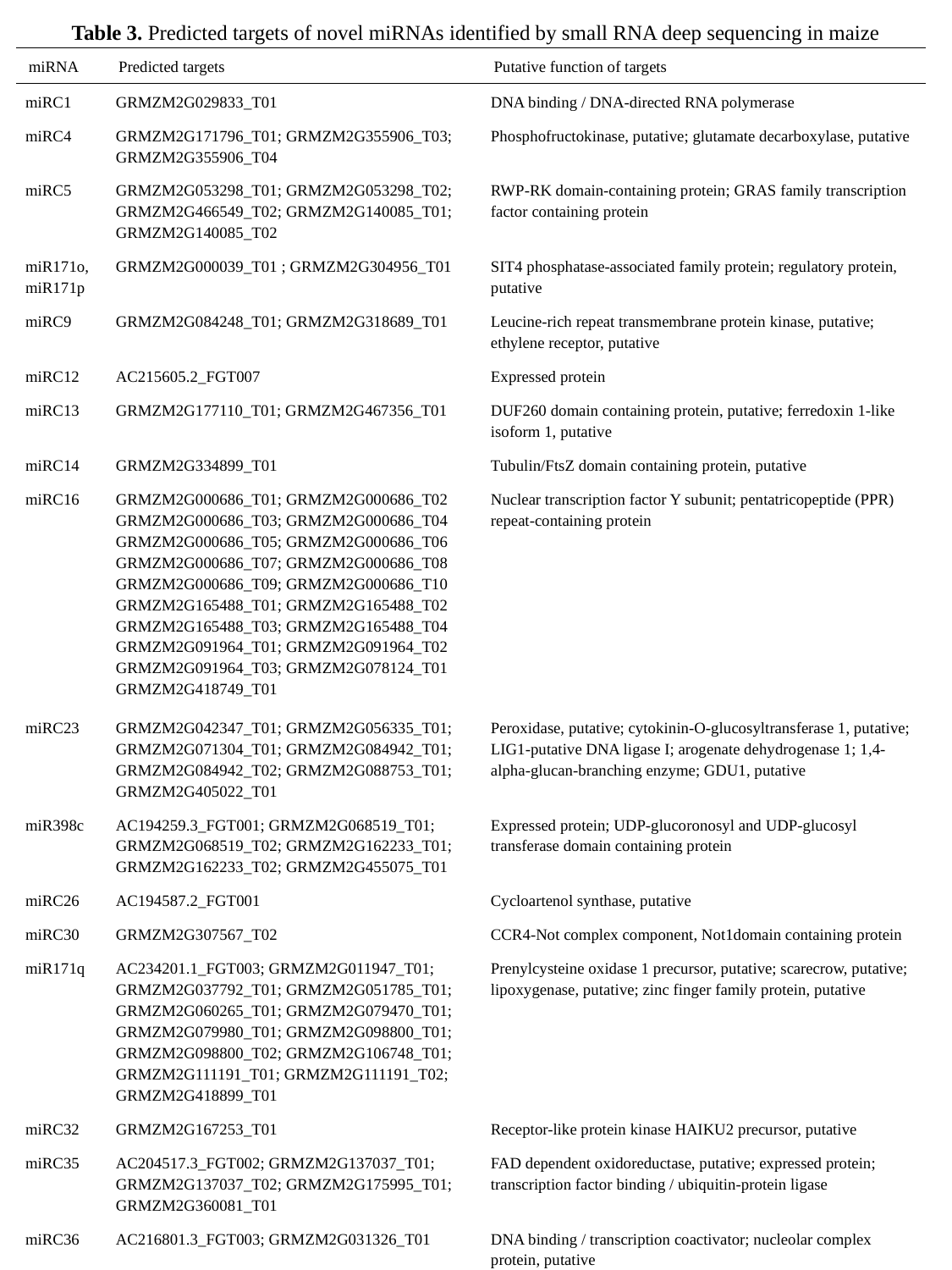

Table 3. Predicted targets of novel miRNAs identified by small RNA deep sequencing in maize
| miRNA | Predicted targets | Putative function of targets |
| --- | --- | --- |
| miRC1 | GRMZM2G029833\_T01 | DNA binding / DNA-directed RNA polymerase |
| miRC4 | GRMZM2G171796\_T01; GRMZM2G355906\_T03; GRMZM2G355906\_T04 | Phosphofructokinase, putative; glutamate decarboxylase, putative |
| miRC5 | GRMZM2G053298\_T01; GRMZM2G053298\_T02; GRMZM2G466549\_T02; GRMZM2G140085\_T01; GRMZM2G140085\_T02 | RWP-RK domain-containing protein; GRAS family transcription factor containing protein |
| miR171o, miR171p | GRMZM2G000039\_T01 ; GRMZM2G304956\_T01 | SIT4 phosphatase-associated family protein; regulatory protein, putative |
| miRC9 | GRMZM2G084248\_T01; GRMZM2G318689\_T01 | Leucine-rich repeat transmembrane protein kinase, putative; ethylene receptor, putative |
| miRC12 | AC215605.2\_FGT007 | Expressed protein |
| miRC13 | GRMZM2G177110\_T01; GRMZM2G467356\_T01 | DUF260 domain containing protein, putative; ferredoxin 1-like isoform 1, putative |
| miRC14 | GRMZM2G334899\_T01 | Tubulin/FtsZ domain containing protein, putative |
| miRC16 | GRMZM2G000686\_T01; GRMZM2G000686\_T02 GRMZM2G000686\_T03; GRMZM2G000686\_T04 GRMZM2G000686\_T05; GRMZM2G000686\_T06 GRMZM2G000686\_T07; GRMZM2G000686\_T08 GRMZM2G000686\_T09; GRMZM2G000686\_T10 GRMZM2G165488\_T01; GRMZM2G165488\_T02 GRMZM2G165488\_T03; GRMZM2G165488\_T04 GRMZM2G091964\_T01; GRMZM2G091964\_T02 GRMZM2G091964\_T03; GRMZM2G078124\_T01 GRMZM2G418749\_T01 | Nuclear transcription factor Y subunit; pentatricopeptide (PPR) repeat-containing protein |
| miRC23 | GRMZM2G042347\_T01; GRMZM2G056335\_T01; GRMZM2G071304\_T01; GRMZM2G084942\_T01; GRMZM2G084942\_T02; GRMZM2G088753\_T01; GRMZM2G405022\_T01 | Peroxidase, putative; cytokinin-O-glucosyltransferase 1, putative; LIG1-putative DNA ligase I; arogenate dehydrogenase 1; 1,4-alpha-glucan-branching enzyme; GDU1, putative |
| miR398c | AC194259.3\_FGT001; GRMZM2G068519\_T01; GRMZM2G068519\_T02; GRMZM2G162233\_T01; GRMZM2G162233\_T02; GRMZM2G455075\_T01 | Expressed protein; UDP-glucoronosyl and UDP-glucosyl transferase domain containing protein |
| miRC26 | AC194587.2\_FGT001 | Cycloartenol synthase, putative |
| miRC30 | GRMZM2G307567\_T02 | CCR4-Not complex component, Not1domain containing protein |
| miR171q | AC234201.1\_FGT003; GRMZM2G011947\_T01; GRMZM2G037792\_T01; GRMZM2G051785\_T01; GRMZM2G060265\_T01; GRMZM2G079470\_T01; GRMZM2G079980\_T01; GRMZM2G098800\_T01; GRMZM2G098800\_T02; GRMZM2G106748\_T01; GRMZM2G111191\_T01; GRMZM2G111191\_T02; GRMZM2G418899\_T01 | Prenylcysteine oxidase 1 precursor, putative; scarecrow, putative; lipoxygenase, putative; zinc finger family protein, putative |
| miRC32 | GRMZM2G167253\_T01 | Receptor-like protein kinase HAIKU2 precursor, putative |
| miRC35 | AC204517.3\_FGT002; GRMZM2G137037\_T01; GRMZM2G137037\_T02; GRMZM2G175995\_T01; GRMZM2G360081\_T01 | FAD dependent oxidoreductase, putative; expressed protein; transcription factor binding / ubiquitin-protein ligase |
| miRC36 | AC216801.3\_FGT003; GRMZM2G031326\_T01 | DNA binding / transcription coactivator; nucleolar complex protein, putative |

## Slide 2
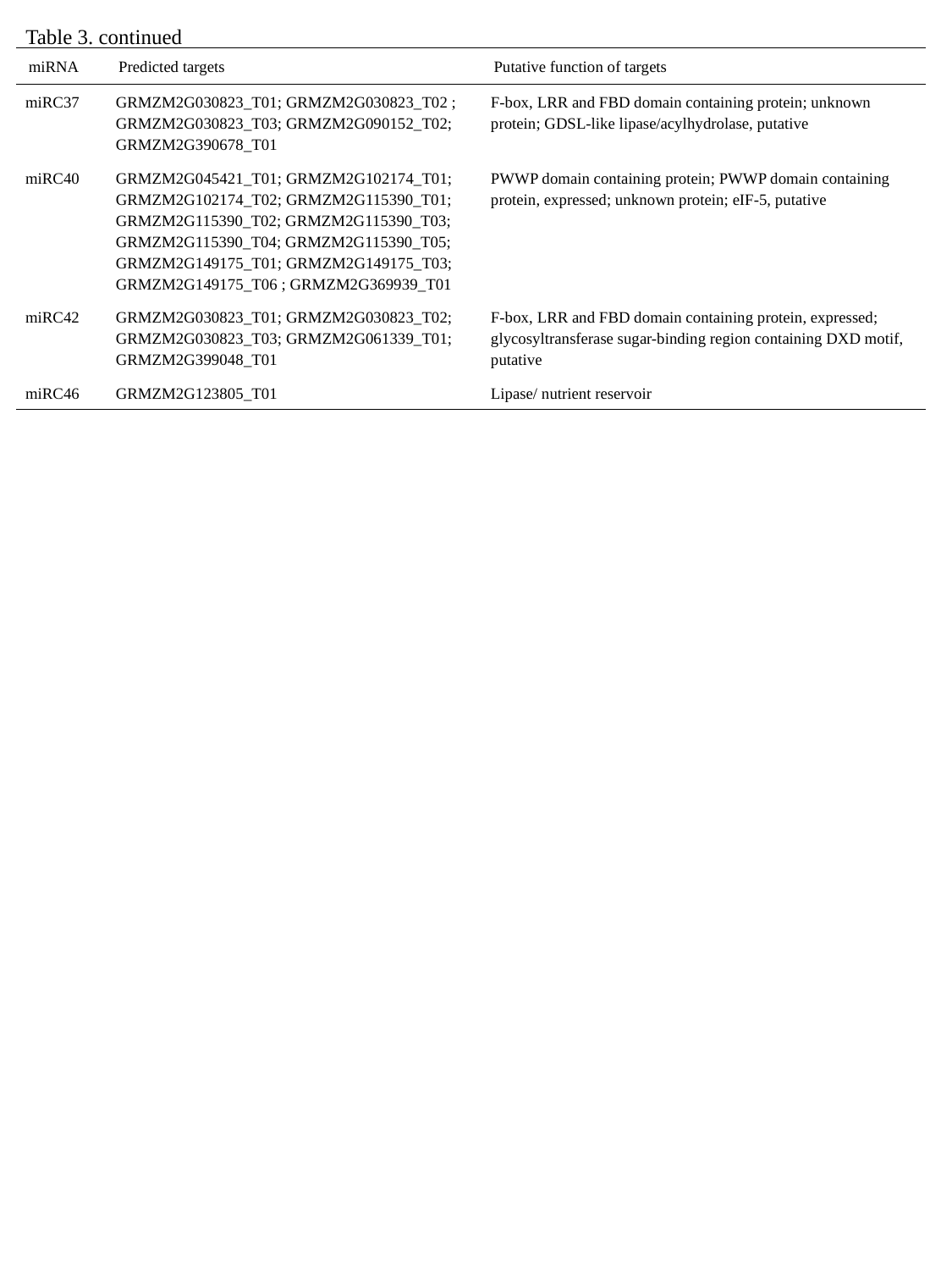

Table 3. continued
| miRNA | Predicted targets | Putative function of targets |
| --- | --- | --- |
| miRC37 | GRMZM2G030823\_T01; GRMZM2G030823\_T02 ; GRMZM2G030823\_T03; GRMZM2G090152\_T02; GRMZM2G390678\_T01 | F-box, LRR and FBD domain containing protein; unknown protein; GDSL-like lipase/acylhydrolase, putative |
| miRC40 | GRMZM2G045421\_T01; GRMZM2G102174\_T01; GRMZM2G102174\_T02; GRMZM2G115390\_T01; GRMZM2G115390\_T02; GRMZM2G115390\_T03; GRMZM2G115390\_T04; GRMZM2G115390\_T05; GRMZM2G149175\_T01; GRMZM2G149175\_T03; GRMZM2G149175\_T06 ; GRMZM2G369939\_T01 | PWWP domain containing protein; PWWP domain containing protein, expressed; unknown protein; eIF-5, putative |
| miRC42 | GRMZM2G030823\_T01; GRMZM2G030823\_T02; GRMZM2G030823\_T03; GRMZM2G061339\_T01; GRMZM2G399048\_T01 | F-box, LRR and FBD domain containing protein, expressed; glycosyltransferase sugar-binding region containing DXD motif, putative |
| miRC46 | GRMZM2G123805\_T01 | Lipase/ nutrient reservoir |
