# Supplementary material for: Cloning and Characterization of Maize miRNAs Involved in Responses to Nitrogen Deficiency
Source: PLoS One. 2012 Jan 3;7(1):e29669. doi: 10.1371/journal.pone.0029669 (PMC3250470; doi:10.1371/journal.pone.0029669)
Supplement: Table S7 — Probes and primers used in this study. (PPT) [file pone.0029669.s007.ppt]

## Slide 1
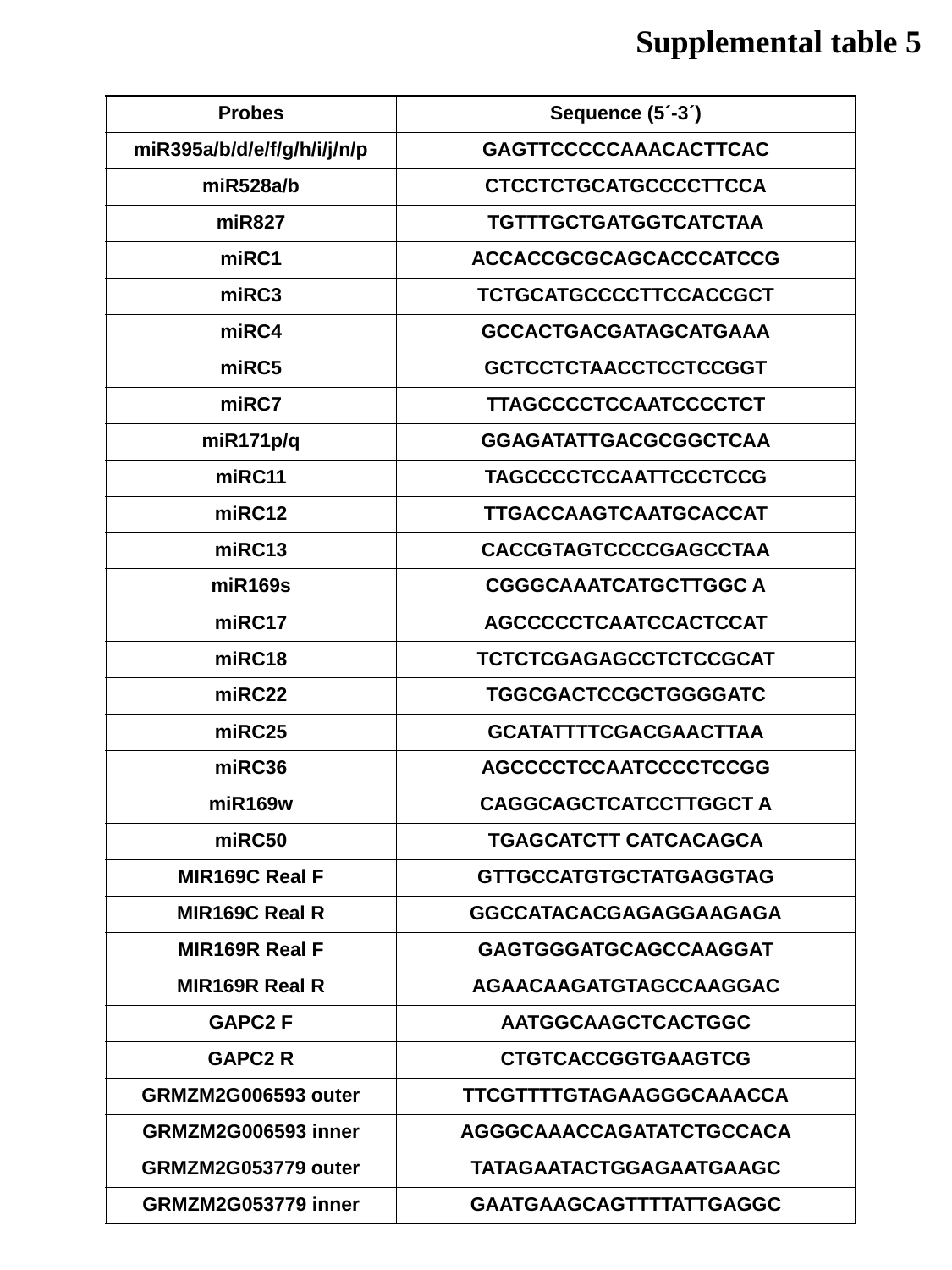

Supplemental table 5
| Probes | Sequence (5´-3´) |
| --- | --- |
| miR395a/b/d/e/f/g/h/i/j/n/p | GAGTTCCCCCAAACACTTCAC |
| miR528a/b | CTCCTCTGCATGCCCCTTCCA |
| miR827 | TGTTTGCTGATGGTCATCTAA |
| miRC1 | ACCACCGCGCAGCACCCATCCG |
| miRC3 | TCTGCATGCCCCTTCCACCGCT |
| miRC4 | GCCACTGACGATAGCATGAAA |
| miRC5 | GCTCCTCTAACCTCCTCCGGT |
| miRC7 | TTAGCCCCTCCAATCCCCTCT |
| miR171p/q | GGAGATATTGACGCGGCTCAA |
| miRC11 | TAGCCCCTCCAATTCCCTCCG |
| miRC12 | TTGACCAAGTCAATGCACCAT |
| miRC13 | CACCGTAGTCCCCGAGCCTAA |
| miR169s | CGGGCAAATCATGCTTGGC A |
| miRC17 | AGCCCCCTCAATCCACTCCAT |
| miRC18 | TCTCTCGAGAGCCTCTCCGCAT |
| miRC22 | TGGCGACTCCGCTGGGGATC |
| miRC25 | GCATATTTTCGACGAACTTAA |
| miRC36 | AGCCCCTCCAATCCCCTCCGG |
| miR169w | CAGGCAGCTCATCCTTGGCT A |
| miRC50 | TGAGCATCTT CATCACAGCA |
| MIR169C Real F | GTTGCCATGTGCTATGAGGTAG |
| MIR169C Real R | GGCCATACACGAGAGGAAGAGA |
| MIR169R Real F | GAGTGGGATGCAGCCAAGGAT |
| MIR169R Real R | AGAACAAGATGTAGCCAAGGAC |
| GAPC2 F | AATGGCAAGCTCACTGGC |
| GAPC2 R | CTGTCACCGGTGAAGTCG |
| GRMZM2G006593 outer | TTCGTTTTGTAGAAGGGCAAACCA |
| GRMZM2G006593 inner | AGGGCAAACCAGATATCTGCCACA |
| GRMZM2G053779 outer | TATAGAATACTGGAGAATGAAGC |
| GRMZM2G053779 inner | GAATGAAGCAGTTTTATTGAGGC |
